# The AAA+ protease ClpXP can easily degrade a 3_1_ and a 5_2_-knotted protein

**DOI:** 10.1038/s41598-018-38173-3

**Published:** 2019-02-20

**Authors:** Elin M. Sivertsson, Sophie E. Jackson, Laura S. Itzhaki

**Affiliations:** 1 Department of Pharmacology, Tennis Court Road, Cambridge, CB2 1PD UK; 2 Department of Chemistry, Lensfield Road, Cambridge, CB2 1EW UK

## Abstract

Knots in proteins are hypothesized to make them resistant to enzymatic degradation by ATP-dependent proteases and recent studies have shown that whereas ClpXP can easily degrade a protein with a shallow 3_1_ knot, it cannot degrade 5_2_-knotted proteins if degradation is initiated at the C-terminus. Here, we present detailed studies of the degradation of both 3_1_- and 5_2_-knotted proteins by ClpXP using numerous constructs where proteins are tagged for degradation at both N- and C-termini. Our results confirm and extend earlier work and show that ClpXP can easily degrade a deeply 3_1_-knotted protein. In contrast to recently published work on the degradation of 5_2_-knotted proteins, our results show that the ClpXP machinery can also easily degrade these proteins. However, the degradation depends critically on the location of the degradation tag and the local stability near the tag. Our results are consistent with mechanisms in which either the knot simply slips along the polypeptide chain and falls off the free terminus, or one in which the tightened knot enters the translocation pore of ClpXP. Results of experiments on knotted protein fusions with a highly stable domain show partial degradation and the formation of degradation intermediates.

## Introduction

**Figure 1:**
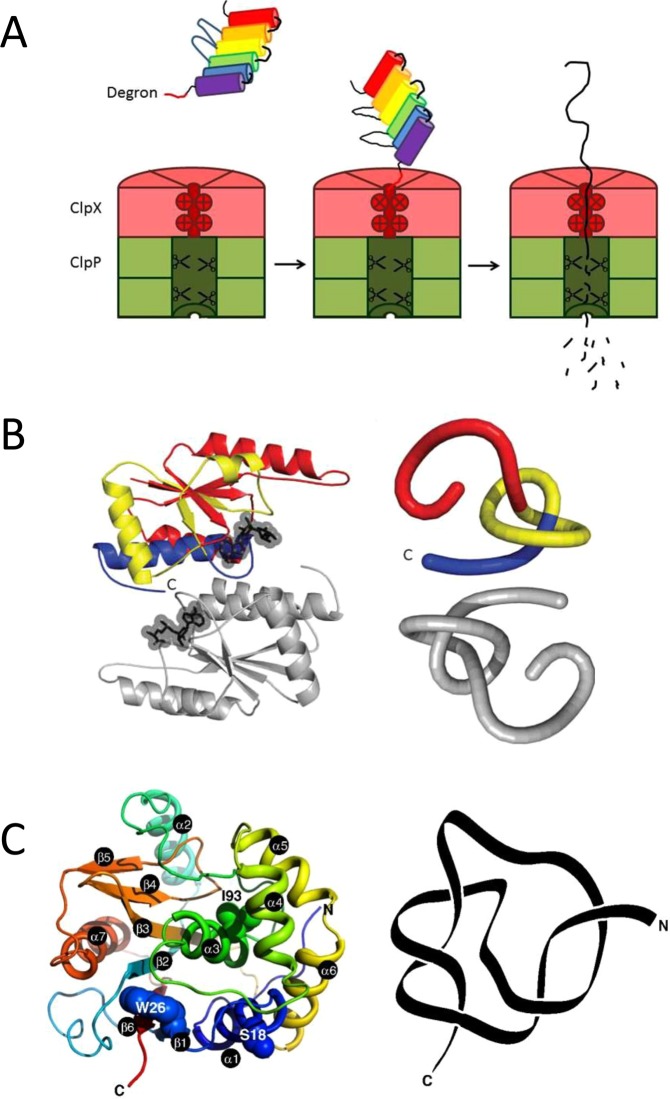
ATP-dependent protease ClpXP and knotted protein substrates. (**A**) Cartoon of protein degradation by ClpXP. (**B**,**C**) Structure and reduced backbone representations of the knotted protein substrates used in this study. (**B**) *E.coli* α/β-knot methyl transferase YbeA (dimeric, PDB 1NS5). Image from Mallam and Jackson^29^. (**C**) Human neuronal ubiquitin C-terminal hydrolase (UCH) UCH-L1 (PDB 2ETL). Image from Andersson *et al*.^29^.

Protein degradation is an essential process in the cell, required to clear faulty or obsolete proteins, recycle amino acids and exert spatial and temporal control over cellular processes. ATP-dependent proteases, such as the 26S proteasome^1^ in eukaryotes and ClpXP in bacteria^2,3^, carry out protein degradation guided by specific degradation signals. ATP-dependent proteases share the same overall architecture resembling a barrel, with a narrow central pore leading to an inner chamber where proteolytic active sites are located^4,5^. According to the model of translocation-coupled unfolding (Fig. 1A), the ATP-dependent protease recognizes a substrate from its degradation signal or degron, engages it in an unstructured region and uses the energy from ATP to mechanically pull the substrate towards the pore opening^6^. As a folded protein is too large to enter the pore, the pulling results in an unravelling of the protein structure at the same time as the polypeptide chain is translocated into the central chamber for proteolytic cleavage^7^. This model explains the finding that local stability is a more important determinant of degradation resistance than is global thermodynamic stability^8^.

Knotted proteins, in which the peptide backbone forms a topological knot, were first discovered in 1994 when Mansfield identified a single protein structure within the PDB with a very shallow knot, carbonic anhydrase^9^. The fact that carbonic anhydrase was unique in having a knotted chain, and that only a few residues needed to pass through a loop to form the very shallow knot, lead Mansfield to conclude that topological knots in proteins, particularly deep ones, were incompatible with protein folding mechanisms^9^. However, in 2000, Taylor identified many more proteins in the PDB with knots, some with deep knots^10^. Since then, an increasing number of knotted proteins have been found in the PDB using computational methods and mathematical knot theory^11,12^. Knots in proteins can be classified based on the number of times the polypeptide chain crosses itself when the chain is projected onto a two-dimensional plane. So far, knots with 3 (trefoil), 4 (figure-of-eight), 5 (Gordian) and 6 (stevedore) crossings have been discovered^13^. In addition, knots have been found with very different depths, depending upon the number of residues that can be removed from each terminus before the knot disappears^14^. The structure that remains after this procedure is referred to as the knotted core. For an up-to-date list of proteins which have either a knotted or slip-knotted structure there is an excellent database – KnotProt^15^. At the time of writing, there are a total of 1539 protein chains with knots in the database, 1040 of which are knotted, and 499 of which form slipknots^15^.

How knotted proteins unfold and fold, and in the latter case how the chain threads through loops to form the different knotted structures observed, has been receiving increasing attention^13,16^. So far, experimental studies have investigated the folding of proteins with 3_1_^17–29^ 5_2_^30–34^ and 6_1_^35^ knotted topologies, and computational studies have tackled 3_1_^36–47^ 5_2_^48,49^ and 6_1_^50^ knotted systems. The first folding studies of knotted proteins were on YibK and YbeA, 3_1_-knotted methyltransferases from *Haemophilus influenzae* and *Escherichia coli*, respectively, both of which have a deep trefoil knot^17,19,20^. Both belong to the α/β-knot methyltransferases (MTases) superfamily of proteins, and like all other family members, they dimerise in the native state, with the knotted region forming a large part of the dimer interface, Fig. 1B ^51^. Here, we use YbeA as a model of a deeply 3_1_-knotted protein.

In addition to the trefoil-knotted proteins, experimental unfolding and folding studies have also been performed on more complex protein knots. The neuronal ubiquitin C-terminal hydrolase UCH-L1 is monomeric and contains a knot with five crossings in a 5_2_ conformation (also called a Gordian knot), Fig. 1C ^52,53^. For more complex knots such as this one, it is helpful to consider not only the knotted core of the chain as a whole, but also the knotting fingerprint of the protein^54^. The knotting fingerprint is represented by a matrix that shows the location and type of any knotted regions formed by the whole chain and sub-regions of the chain. Almost the entire chain of UCH-L1 forms a 5_2_ knot. If 4 residues are removed from the C-terminus the 5_2_ knot is abolished, but a 3_1_ knot remains until a further 56 residues are removed. In contrast, if residues are progressively removed from the N-terminal end, only 5 residues need to be removed before the chain becomes completely unknotted^54^. Despite its complex topology, UCH-L1 can be reversibly unfolded *in vitro* with chemical denaturants^31,55^ and experimental unfolding and refolding studies have elucidated many details of its folding pathway^32,34,55^. Of particular relevance to this study, recently optical tweezers were used to investigate the mechanical unfolding and subsequent refolding of UCH-L1^33^. Numerous intermediate states were observed in both unfolding and refolding experiments illustrating that the energy landscape for folding of UCH-L1 is very complex.

Both experimental and computational approaches have been used to investigate the effect of knotted topologies on the mechanical unfolding properties of the proteins. Simulations of two proteins having similar structures where one is knotted and the other is not showed that a knot can impart additional stability towards mechanical force^36^. In this case, the knot was observed to move during pulling simulations, preferentially ending up at positions of the chain with sharp turns, usually at proline or glycine residues^36,56^. Computational approaches have also been employed to investigate the probability of untying a knot in a protein and its dependence on pulling site, pulling speed and temperature^57^. AFM and more recently optical tweezers experiments have investigated the force-induced unfolding of a number of different knotted proteins. AFM experiments on phytochrome c, a protein with a figure-of-eight^41^ knot, first established that mechanical pulling leads to unfolding and then knot tightening demonstrating that very tight knots which are densely packed can form even at biologically relevant forces^58^. In this case, the tightened knot comprised of 17 residues^58^. More recently the Li group mechanically unfolded a slip-knotted protein to a denatured state containing a trefoil knot and showed that the tightened knot comprised of 13 residues^59^. Most recently, a study using optical tweezers to force unfold the 5_2_-knotted protein UCH-L1 into 5_2_, 3_1_ and unknotted denatured states was published^33^. The trefoil-knotted state comprised of 12-13 residues in agreement with the Li study, and the 5_2_ -knotted denatured state showed complex behaviour in which the knot first tightens to a state involving some 40 residues but upon the application of higher forces further tightens to a state comprising of 23 residues^33^.

The biological function of protein knots is not known, however, it has been hypothesised that the presence of a knot in a polypeptide chain may render a protein resistant to degradation by ATP-dependent proteases^34^. A number of published *in silico* studies of knotted proteins suggest this may be possible. For example, the results of simulations which mimic the pulling and translocation of a trefoil-knotted protein through a narrow pore have shown that the knot either tightens and blocks further translocation or it slides off the free end of the polypeptide chain as the protein is pulled through the pore depending upon the force used^60–62^. Recently, computational studies have simulated the translocation of knotted proteins through the pore in a simple model of a cellular degradation machine^63^. In this case, the simulations showed that the presence of a knot can hinder or even jam translocation^63^. More importantly, a recent experimental study showed that a shallow 3_1_-knotted protein MJ0366 could be degraded by ClpXP, and that a number of C-terminal fusions of MJ0366 with GFP resulted in stalling and partial degradation^64^. However, another recent study showed that when the degradation tag was attached to the C-terminus of 5_2_-knotted proteins from the ubiquitin C-terminal hydrolase family (UCH) little degradation was observed leading the authors to conclude that this class of knotted protein has unprecedented mechanostability^65^.

Here, we examine the ClpXP-catalysed degradation of two different knotted proteins, the deeply knotted YbeA which contains a 3_1_ knot and the larger and more complex 5_2_-knotted protein UCH-L1. In contrast to previous studies, we initiate degradation from both N- and C-termini, we use destabilised variants of UCH-L1 to establish the role of local stability versus the knotted structure on degradation rates, and also we investigate the degradation of 3_1_- and 5_2_-knotted proteins fused to a small but highly stable domain that we show can withstand the mechanical unfolding force of ClpXP and therefore degradation. For the 3_1_-knotted YbeA we observe results similar in some ways to those recently published on MJ0366: the ClpXP degrades the protein with relative ease but when a super-stable ThiS domain is fused at the opposite termini to the degradation tag, we see partial degradation, and the formation of a degradation intermediate. For the 5_2_-knotted UCH-L1, we initiate degradation from both N- and C-termini and find significant differences in degradation rates. Using a number of destabilised variants, we can attribute this to differences in local stability rather than the knotted state of the protein, demonstrating for the first time that ClpXP can easily degrade larger more complex knotted structures as well as smaller 3_1_-knotted proteins. Thus, we demonstrate that the origin of the “unprecedented mechanostability” stated in previous work is local stability of the β-sheet structure and is not associated with the 5_2_ knot. With UCH-L1 fusions, we also see partial degradation similar to the results for the 3_1_ systems. The fact that even large knotted chains can enter ClpXP points to the remarkable flexibility of its central pore.

## Results

### Degradation of a trefoil-knotted protein

First, we studied the degradation of the 3_1_-knotted methyltransferase YbeA, which contains a deep trefoil knot, with 70 and 34 residues lying to the N- and C-terminus of the knotted core, respectively (Fig. 1B). This is in contrast to the shallow trefoil knot of MJ0366 previously studied^64^ where the numbers are 10 and 6 residues for the distance of the knot from the N- and C-terminus, respectively. YbeA was fused with the 11-amino acid ssrA degron^66,67^ at the C-terminus to produce YbeA-ssrA. To confirm that the knot in YbeA is still present after the addition of the ssrA-tag at the C-terminus, analytical gel filtration of YbeA-ssrA was performed (Fig. S1A). YbeA eluted at a volume of 16.3 ml, which corresponds to a *K*_av_ of 0.52 and a molecular mass of 38.9 kDa. This value is close to the calculated molecular mass of a dimer, 36.9 kDa. As the dimer interface is largely made up of the knotted region^18^, the results suggest that the knot remains intact in YbeA-ssrA.

**Figure 2:**
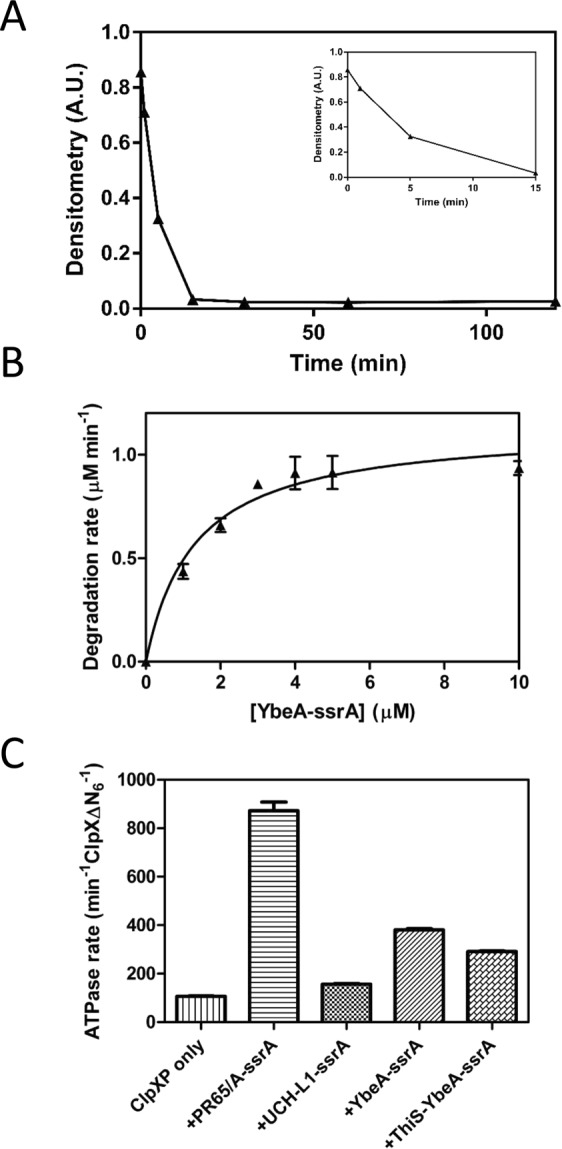
Degradation of the trefoil knotted protein YbeA-ssrA by ClpXP. (**A**) Degradation of YbeA-ssrA (10 µM) by ClpXP (0.5 µM ClpXΔN_6_, 1 µM ClpP_14_), monitored by SDS-PAGE and densitometry. The reaction contained 4 mM ATP with regeneration system. Inset shows the first 15 minutes of the reaction. (**B**) Michaelis-Menten plot of YbeA-ssrA degradation. Initial rates of degradation were calculated from loss of gel band density of SDS-PAGE gels. Error bars represent SEM of triplicate measurements. The data are fitted to the Michaelis -Menten equation (v = V_max_ [S]/(*K*_M_ + [S]), and the rate constant for degradation was calculated as *k*_deg_ = *V*_max_/[ClpX_6_]. (**C**) Steady state ATP hydrolysis rate of ClpXP (0.4 µM ClpXΔN_6_, 2.2 µM ClpP_14_) at 30 °C, alone or in the presence of ssrA-tagged substrate (10 µM). Error bars represent the SEM of at least three measurements.

YbeA-ssrA was degraded rapidly by ClpXP (Figs 2A and S2A). The initial degradation rate at different concentrations of YbeA-ssrA was measured and the data were fitted to the Michaelis-Menten equation to extract the kinetic parameters (Fig. 2B, *k*_deg_ = 2.3 ± 0.1 min^−1^ ClpX_6_^−1^, *K*_M_ = 1.3 ± 0.3 µM). These numbers are comparable to those reported for the degradation of a series of variants of titin by ClpXP (ranging from 0.25 < *k*_deg _ < 5 min^−1^ ClpX_6_^−1^ and 1.1 < *K*_m_ < 2.9 μM)^68^, and that recently reported for the 3_1_-knotted MJ0366 (*k*_cat_ = 3 min^−1^)^64^. The rate of ATP-hydrolysis by ClpXP was moderately stimulated during the degradation process (Fig. 2C).

#### Degradation of a trefoil-knotted fusion protein

**Figure 3:**
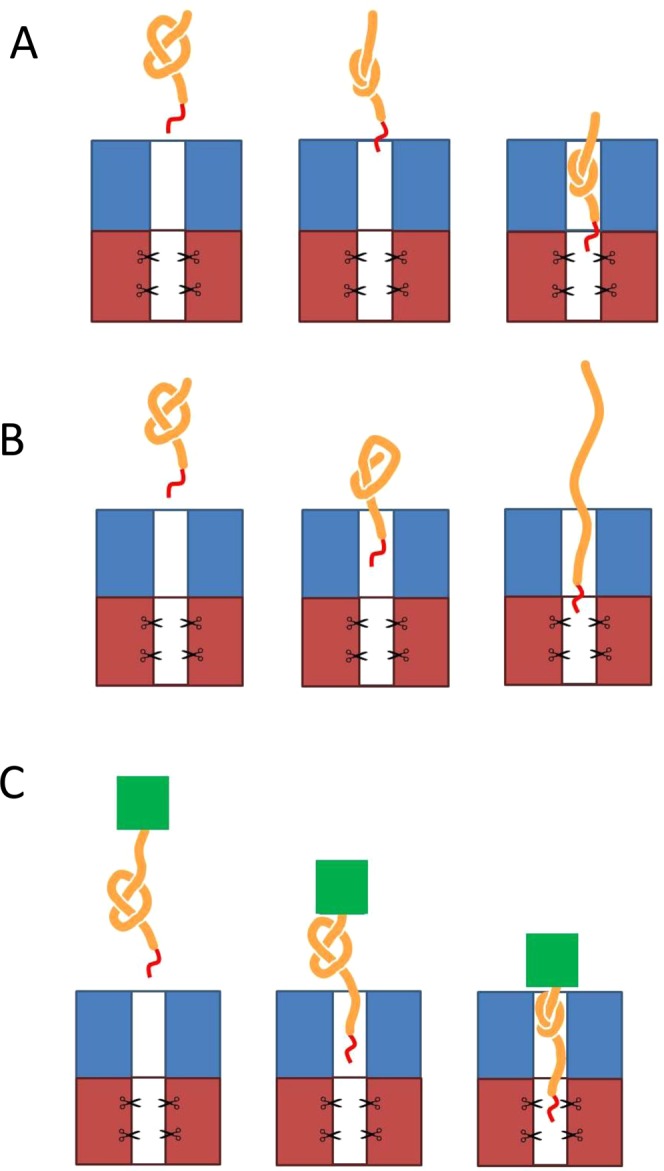
Models of ClpXP-catalysed degradation for trefoil knotted proteins. Trefoil knotted protein (orange), ssrA-tag (red), ATP-dependent protease (blue/red), highly stable ThiS ‘plug’ domain (green). (**A**) Knot tightening and translocation into the degradation machinery. (**B**) Knot slips off the end of the polypeptide chain as the protein is unfolded and translocated into ClpXP. (**C**) Knot slipping along the chain before tightening and being blocked by the addition of a highly stable ThiS domain.

**Figure 4:**
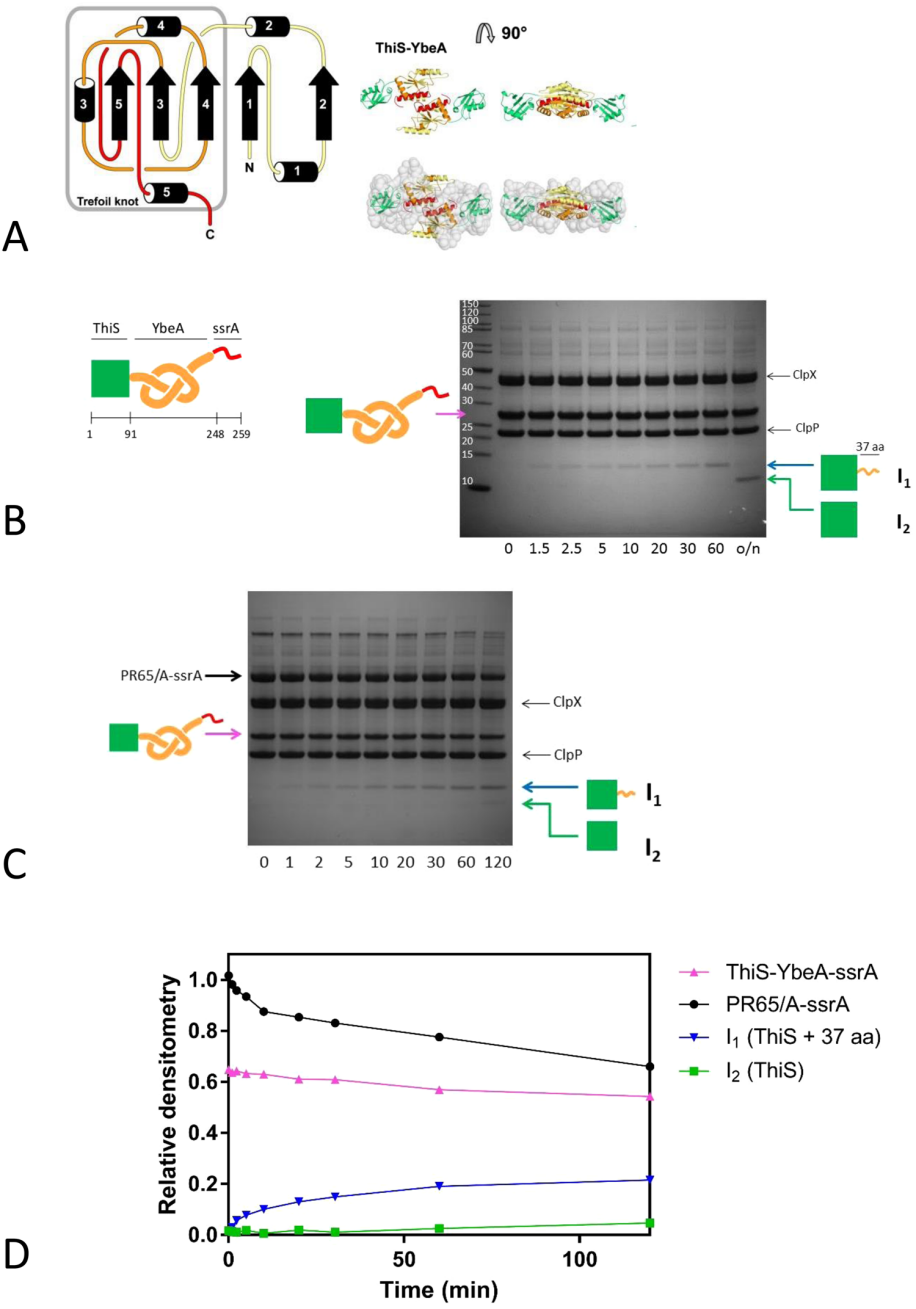
Degradation of the knotted fusion protein ThiS-YbeA. (**A**) Modelled structure of ThiS-YbeA, from Mallam *et al*.^21^. Left, topology of YbeA. The knotting loop is shown in orange, with the knotted chain in red. Right, modelled structure of ThiS-YbeA. YbeA is coloured as in the topology diagram, ThiS is in green. (**B**) Degradation of ThiS-YbeA-ssrA. Left, schematic representation of ThiS-YbeA-ssrA: ThiS (green square), YbeA (orange knot) and ssrA-tag (red line), with residue numbering. Right, degradation of ThiS-YbeA-ssrA (10 µM) by ClpXP monitored by SDS-PAGE. (**C**) Degradation competition experiment with equimolar amounts of PR65/A-ssrA and ThiS-YbeA-ssrA (5 µM each), monitored by SDS-PAGE and (**D**) subsequent densitometry. The reactions contained 0.5 µM ClpXΔN_6_, 1 µM ClpP_14_ and 4 mM ATP with regeneration system. Times in minutes indicated below the gel. Arrows indicate position of protein species as confirmed by gel band mass spectrometry analysis: PR65/A-ssrA (black circles), full-length ThiS-YbeA-ssrA (pink triangles), degradation intermediate I_1_ (ThiS + 37 residues, blue inverted triangles), degradation intermediate I_2_ (ThiS, green squares). In theory, the total amount of ThiS-YbeA-ssrA, I_1_ and I_2_ should equal 1, but it is greater than 1. We think that this discrepancy lies in the fact that the band for the full-length ThiS-YbeA-ssrA overlaps with the band in the gel from the ATP regeneration system, thus leading to some inaccuracies in the exact quantities of the species present. As MS data clearly show I_1_ and I_2_ come from the parent ThiS-YbeA-ssrA construct, we are not too concerned about this small difference.

Is YbeA-ssrA degraded by ClpXP because the knot is small enough to fit through the translocation pore, Fig. 3A? Alternatively, does the knot slide off the free N-terminal end of the protein as its C-terminus is pulled through the ClpXP pore, Fig. 3B? To further investigate these questions, we made a ssrA-tagged fusion protein of YbeA and the small, very stable protein ThiS from the thermophile *Archaeoglobus fulgidus*^69^ (Fig. 4A). It has been shown that despite an additional ThiS domain at either the N- or C-terminus of YbeA, the fusion proteins can refold into their native knotted structures both after urea denaturation^69^ and after *in vitro* translation^70^. Here, we used a construct in which the ThiS domain was fused to the N-terminus of YbeA with a ssrA tag at its C-terminus (ThiS-YbeA-ssrA). During mechanical pulling of the protein at the C-terminus by the ClpXP machine, ThiS is expected to function as a ‘molecular plug’ blocking the N-terminus of YbeA and preventing the knot from sliding off the end of the chain (Fig. 3C), similar to the use of GFP fusions in the recent study on the shallow 3_1_-knotted MJ0366^64^. The fusion protein, ThiS-YbeA-ssrA ran as a dimer in size-exclusion chromatography (Fig. S1B), suggesting that it had assumed its native knotted state.

ThiS-YbeA-ssrA displayed complex degradation behaviour. There was loss of signal corresponding to the full-length protein and a gradual appearance of a smaller species in the gel when the reaction time points were analysed by SDS-PAGE (Fig. 4B). This small species was processed into an even shorter form after overnight incubation, Fig. 4B. Mass spectrometric analysis of the respective gel bands showed that the larger species (referred to as degradation intermediate I_1_) is a truncation of the fusion protein ending in position 128, Fig. S10A,B. As ThiS is 91-residues long and there is a short GlySer linker, this species corresponds to ThiS with an additional 37 residues (35 from YbeA and two from the GlySer linker) at the C-terminus. These 37 residues are consistent with the 30–37 residues known to span the distance from the ClpX opening to the proteolysis site in ClpP^71,72^. The smaller species (referred to as degradation intermediate I_2_) is a further truncation ending between positions 95 and 105, Fig. S10C,E, which is therefore likely to be ThiS on its own without the pore-spanning tail. We attribute this further processing to a contaminating protease as the I_1_ intermediate does not have a ssrA tag. The ATPase rate during degradation of ThiS-YbeA-ssrA was stimulated moderately (291 ± 3 min^−1^ ClpX_6_^−1^), though lower than compared to YbeA-ssrA (380 ± 5 min^−1^ ClpX_6_^−1^) (Fig. 2C).

As the loss of ThiS-YbeA-ssrA is hard to observe, we repeated the degradation experiment in the presence of a competitive substrate, PR65/A-ssrA, which is a topologically simple helical repeat protein that we know is easily degraded by ClpXP (Fig. S4). In this way, we ascertained that ThiS-YbeA-ssrA did engage with the ClpXP machinery (Figs 4C and S3A) as the degradation of PR65/A-ssrA was slower in the presence of This-YbeA-ssrA. The degradation intermediates appeared with time as expected and, in this experiment, it was clearer that the larger intermediate I_1_ was gradually being processed into the smaller intermediate I_2_ as expected (Fig. S3A). Densitometric analysis of the gel (Fig. 4D) showed that some of ThiS-YbeA-ssrA as well as some PR65/A-ssrA was degraded, but neither protein was degraded to completion. We repeated the experiment using three times the concentration of enzyme in the degradation assay. Densitometric analysis of SDS-PAGE gels (Fig. S3B,C) confirmed that the ratio of the I_1_ (or I_2_) band at maximum intensity and the ClpP band was the same in this experiment as when using the lower enzyme concentration, indicating that the increase in the amount of enzyme has resulted in a proportional increase in the amount of intermediate. Moreover, a larger fraction of full-length ThiS-YbeA-ssrA was degraded before the reaction came to a halt. Degradation-tagged ThiS on its own (ThiS-ssrA) was not degraded by ClpXP (Fig. S4).

### Degradation of a more complex 5_2_-knotted protein by ClpXP

As our results, and those of the recent study by San Martin *et al*.^64^ showed that ClpXP is able to degrade a 3_1_-knotted protein with relative ease, we moved on to study the degradation of a protein with a larger, more complex knot. UCH-L1 (ubiquitin C-terminal hydrolase L1) has a 5_2_ knot that is shallow from the N-terminal end (only 5 residues need to be deleted to result in an unknotted chain) but deeper from the C-terminus (removal of some 4 residues results in formation of a deep trefoil knot which requires further deletion of more than 56 residues for the chain to become completely unknotted) (Fig. 1C). Recently, the Hsu group has shown that members of the UCH family including UCH-L1 withstand degradation by ClpXP when the ssrA degron is placed at the C-terminus^65^. Here, we carried out similar experiments and UCH-L1 was fused with the 11-amino acid ssrA degron at the C-terminus, but we additionally made a second variant in which we attached the ssrA degron at the N-terminus of UCH-L1 using a covalent chemical linkage strategy^8^.

#### Degradation of 5_2_-knotted UCH-L1

**Figure 5:**
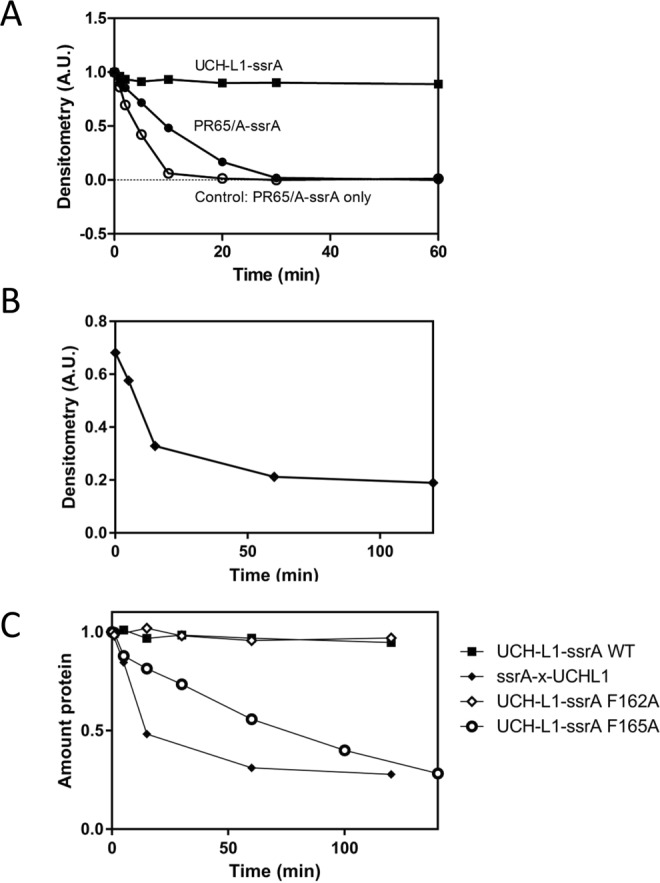
Degradation of N- and C-terminally ssrA tagged variants of the 5_2_-knotted protein UCH-L1 by ClpXP. (**A**) ClpXP degradation competition experiment with equimolar amounts of UCH-L1-ssrA (closed squares) and PR65/A-ssrA (closed circles) (5 µM each). Control reaction with PR65/A-ssrA (5 µM) without competitor shown as open circles. (**B**) Degradation of UCH-L1 with a crosslinked N-terminal ssrA-tag (ssrA-x-UCH-L1, 10 µM, closed diamonds). All reactions contained 0.5 µM ClpXΔN_6_, 1 µM ClpP_14_ and 4 mM ATP with regeneration system and were monitored by SDS-PAGE and densitometry. (**C**) Degradation of UCH-L1-ssrA variants by ClpXP (0.5 µM ClpXΔN_6_, 1 µM ClpP_14_) monitored by SDS-PAGE and densitometry: UCH-L1-ssrA wild type (10 µM, solid squares); F165A (9 µM, open circles), F162A (10 µM, open diamonds), ssrA-x-UCH-L1 (10 µM, closed diamonds). The reactions contained 4 mM ATP with a regeneration system.

First, we studied the degradation of UCH-L1-ssrA where the degron is located at the C-terminus of the knotted protein. We obtained very similar results to the Hsu group and did not detect any significant degradation of UCH-L1-ssrA by ClpXP over 60 mins, Fig. 5A. In a competition experiment with equimolar concentrations of UCH-L1-ssrA and the topologically simple helical repeat protein PR65/A-ssrA (Fig. 5A), the presence of UCH-L1-ssrA slowed down the degradation of PR65/A-ssrA about two-fold. This result indicates that ClpXP was able to bind to UCH-L1-ssrA but not degrade it.

ClpXP displays a basal rate of ATP hydrolysis even when not engaged in protein degradation and in the presence of ssrA-tagged substrate, the ATPase rate is stimulated^73^. The rate of ATP hydrolysis of ClpXP was not stimulated to any greater degree by the addition of UCH-L1-ssrA compared to the stimulation upon addition of PR65/A-ssrA (Fig. 2C), suggesting that although UCH-L1-ssrA engages with ClpXP it cannot be unfolded and translocated by it.

The 5_2_ knot in UCH-L1 knot is located between residues 5-219, whereas a 3_1_ knot exists between residues 5-163. Thus, deletion of a much smaller length of chain is required to fully unknot the protein from the N-terminal end (5 residues) than from the C-terminal end (more than 60 residues). Therefore, we hypothesized that the terminus at which degradation is initiated may make a difference for knot unfolding. To examine this question, a mutant of UCH-L1 with a single cysteine located close to the N-terminus (named Q2C) was employed. Circular dichroism showed that the native structure of the Q2C mutant is unperturbed (Fig. S5A) and that its thermal stability is identical to that of wild-type UCH-L1-ssrA (Fig. S5B).

A synthetic ssrA peptide was chemically crosslinked to the cysteine of the mutant protein^8^. The yield from the crosslinking reaction was approximately 50% (Fig. S6). The crosslinked species (ssrA-x-UCH-L1) was purified by nickel affinity using a hexahistidine tag in the peptide sequence prior to the degron sequence and used in degradation experiments with ClpXP. In contrast to the results obtained for UCH-L1-ssrA, there was visible degradation of ssrA-x-UCH-L1 in the presence of ClpXP and ATP (Figs 5B and S2C). The half-life was estimated to be 10 minutes for 10 µM substrate protein. Low amounts of non-crosslinked protein without the ssrA-tag, visible as a weak band of slightly lower molecular weight than ssrA-x-UCH-L1, were present but not degraded by ClpXP, Fig. S2C.

#### UCH-L1 mutants

C-terminally tagged UCH-L1-ssrA was not noticeably degraded by ClpXP, Fig. 5A. N-terminally tagged ssrA-x-UCH-L1, on the other hand, was degraded by ClpXP, Fig. 5B. The fact that the C-terminal ssrA-tag is attached directly to β-strand 6, which is located at the centre of the core β-sheet structure, may explain the resistance of UCH-L1-ssrA to ClpXP-induced degradation. UCH-L1 unfolding by chemical denaturants proceeds via a stable intermediate, where the central part of this β -sheet remains highly structured^31^. It has previously been shown that the local structure adjacent to the degradation signal is more important than the overall thermodynamic stability of a protein in determining its resistance to degradation by ATP-dependent proteases^71^. It is possible that the stability of the secondary structure of the region where the tag is attached, rather than the overall knotted topology of the protein as a whole, may be a more important factor in the resistance of UCH-L1-ssrA to degradation by ClpXP. To test this possibility, we designed UCH-L1 mutants where the β-sheet is destabilised by the replacement of a large hydrophobic residue with an alanine. Mutants UCH-L1-ssrA F162A and UCH-L1-ssrA F165A were selected based on their theoretical destabilisation of 5.4 kcal mol^−1^ and 5.9 kcal mol^−1^, respectively (calculated using FoldX^74,75^). The thermodynamic stability of these different substrates was compared in a thermal melt assay, monitored by far-UV circular dichroism (Fig. S5C). Although UCH-L1-ssrA F162A was somewhat stabilised by the mutation, UCH-L1-ssrA F165A was very destabilised. A UCH-L1 functional assay, which measures the hydrolysis of the fluorescent substrate Ub-AMC (Fig. S7), showed that both mutants retained enzymatic activity, suggesting that their native structures, and therefore knotted state, are unaltered.

The somewhat stabilised UCH-L1-ssrA F162A was not degraded by ClpXP (Fig. 5C). However, the significantly destabilised UCH-L1-ssrA F165A was degraded by ClpXP (Fig. 5C), suggesting that the reduced thermodynamic stability of the β-sheet is sufficient to allow degradation.

Lastly, we examined an N-terminal fusion of UCH-L1 and the extremely stable ThiS domain. The fusion protein ThiS-UCH-L1-ssrA also displayed the appearance of a degradation intermediate (referred to as I_TU_) over time (Fig. S8). Mass spectrometry detected three fragments in the gel band, all C-terminal truncations starting at residue 1 and ending at residues 140, 142 and 143, respectively (Fig. S11). In the ThiS-UCH-L1-ssrA construct, the ThiS protein ends at position 109. That is, the detected intermediates are ThiS with a tail of 32–35 residues. The tightened knot in UCH-L1 is estimated from AFM studies to comprise roughly 40 residues up to 40 pN of force and is further tightened to 23-24 residues at higher forces^33^. Thus, the observed tail of ThiS-UCH-L1-ssrA is not long enough to accommodate a 5_2_ knot, even if fully tightened. In fact, the tail is just long enough to span the distance from the ClpX opening to the ClpP proteolytic sites provided.

## Discussion and Conclusions

A significant number of knotted proteins have now been identified with differing knot complexities and sizes^13,15^. Mechanical unfolding studies have established that applying force to the N- and C-termini of these proteins results in unfolding and the subsequent formation of a tight knot in the denatured state^33,58,59^. The size of the tightened knot varies depending upon the knot type and for 3_1_-knotted proteins has been shown to comprise approximately of 12–14 residues and simulations suggest that tightened trefoil knots in proteins have a radius of gyration of around 7 Å^33,59,76,77^. In contrast, 4_1_-knotted proteins have larger knots comprising some 15-16 residues^58^ and the even larger 5_2_-knotted protein UCH-L1 shows complex behaviour and initially adopts a tightened knot comprising around 40 residues but tightens further to a smaller knot comprising some 23 residues at high forces^33^. Recently, San Martin and co-workers showed that, under some conditions the ClpXP machinery could easily degrade the shallow 3_1_-knotted protein MJ0366, whereas under other conditions the knot impaired degradation of specific multi-domain protein constructs^64^. In addition, another recently published paper reported that the ClpXP machinery only degrades a class of 5_2_-knotted proteins with unprecedented slow kinetics which the authors attribute to the remarkable mechanostability of the protein due to the large complex 5_2_-knot. However, in that study degradation was only initiated from the C-terminus^65^.

In this study, we have addressed the question of whether the ClpXP machine can degrade 3_1_- (trefoil) knotted proteins which contain very deep knots in their chains and also 5_2_ knotted proteins if degradation is initiated from either N- or C-termini, and if the local stability near the degradation tag is reduced. Rapid degradation of the C-terminally ssrA-tagged YbeA was observed (Fig. 2) similar to the results on a C-terminally ssrA-tagged MJ0366, demonstrating that trefoil-knotted proteins, if they are not fused to any other domains, can be easily degraded by ClpXP and that it does not matter whether the 3_1_ knot is shallow or, as we have shown here, deep. In the case of the rapid degradation of YbeA-ssrA by ClpXP we can imagine two different scenarios (we ignore the possibility that partial degradation occurs followed by release, unknotting and then rebinding and further degradation as the protein loses its degron as soon as degradation begins). Scenario (i) Once the ClpXP machinery has unfolded the YbeA domain, the knot is displaced along the chain as translocation takes place until it falls off the free end (Fig. 3B) and (ii) the knot is sufficiently small to be translocated through the ClpXP pore and is thus degraded when it reaches the proteolytic domain (Fig. 3A).

In order to investigate whether scenario (i) or (ii) takes place, we engineered a variant of YbeA-ssrA in which a small but highly stable domain, ThiS, was fused to the N-terminus of YbeA. We tested ThiS on its own with a C-terminal degron, ThiS-ssrA, and found that it is not degraded by ClpXP to any degree (Fig. S4). We assume that this is due to the very high kinetic and thermodynamic stability of ThiS (Fig. S5D) and^69,70^. ThiS-YbeA-ssrA showed complex behaviour in the degradation assays (Fig. 4) and the results shed light on whether scenario (i) or (ii) is occurring.

**Figure 6:**
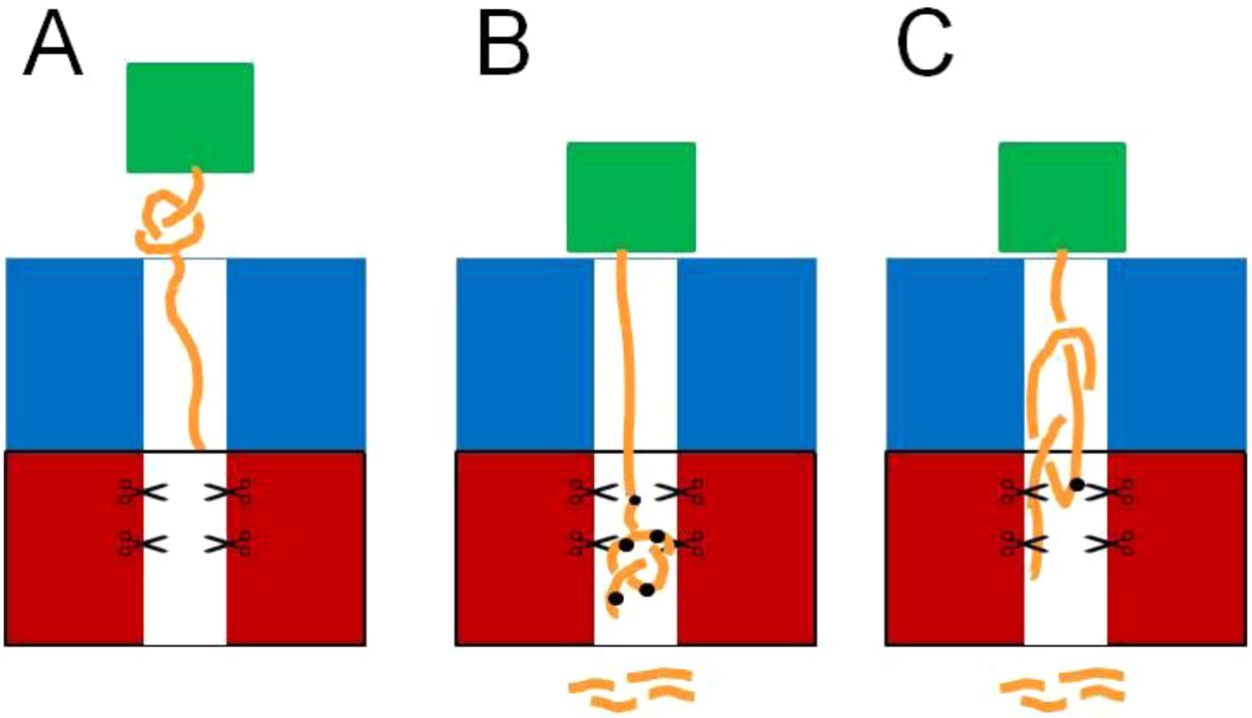
Models of the ClpXP-catalysed degradation of knotted fusion proteins. Trefoil knotted protein (orange), ATP-dependent protease (blue/red), highly stable ThiS ‘plug’ domain (green). (**A**) Knot tightening and abutting the stable ThiS domain outside of the ClpXP machinery. In this model, once the YbeA domain has been unfolded, the knot slips along the chain as translocation takes place until it reaches the ThiS domain at which point it can go no further. The knot does not enter the translocation pore. (**B**) The tightened knot enters the translocation pore and when it reaches the proteolytic active sites it is hydrolysed. The chain continues to translocate and be degraded until the stable ThiS domain abuts the surface of ClpXP. (**C**) The tightened knot slips along the polypeptide chain until it can slip no further as it abuts the ThiS domain. At this point, the knot can enter the translocation pore and part of the knotted region reaches the proteolytic sites and is hydrolysed.

Scenario (i): In this case, ClpXP engages with the fusion protein through the ssrA degron at the C-terminus and unfolds the YbeA domain retaining the 3_1_ knot. It subsequently translocates and degrades the chain with the knot slipping along the chain until it abuts the very stable ThiS domain (Fig. 6A). At this point, the knot should become tightened and the degradation machinery stall until the chain dissociates from it. If this happens, a degradation intermediate corresponding to the ThiS domain plus approximately 50 residues (the length of the tightened knot plus the length of chain that spans ClpXP from the pore opening to the proteolytic sites). We do observe a degradation intermediate but it is significantly shorter than this, suggesting that this scenario is incorrect. We can also rule out similar scenarios where the knot tightens before it has reached the ThiS domain (Fig. S9) that would result in degradation intermediates of even longer length.

In scenario (ii), after the ClpXP machinery engages with the C-terminal ssrA tag and unfolds the YbeA domain, the knot (which we assume will be tightened at some point in the chain) can translocate into the pore and to the proteolytic sites (Fig. 6B). Translocation and degradation will continue until the stable folded ThiS domain abuts the ClpXP and then will stop (Fig. 6B). In this case, one would expect to observe a degradation intermediate that is the length of ThiS plus approximately 37 residues, the amount of chain required to span the non-proteolytic domain of ClpXP. This is what we observe (Figs 4B and S3) and this result together with recent results on the shallow 3_1_- knotted MJ0366 suggests that the pore of ClpXP is large and flexible enough to accommodate a tightened 3_1_ knot. This is perhaps not surprising given that it has been shown in computational studies that the diameter of a tightened 3_1_- knot is approximately 14 Å^76,77^ similar to the diameter of the translocation pore of ClpXP^78^. In addition, it has been shown that ClpXP can translocate disulphide-linked chains, meaning that at least two, or even three, polypeptide chains can fit in the ClpXP pore at the same time^73^. Indeed, the 26S proteasome is able to degrade disulfide-linked substrates with at least three, possibly up to five, chains passing through the translocation channel at once, albeit with lower degradation rate and efficiency^79^.

ClpXP was shown to degrade the trefoil-knotted protein YbeA with relative ease, perhaps unsurprising given that it has been estimated that the ClpXP opening is able to accommodate up to three disulfide-bonded chains that would require an opening of at least 20–25 Å. With an estimated diameter of approximately 14 Å, a trefoil knotted chain can translocate into the ClpXP pore and be degraded, as we and others^64^ have observed. Recently, other groups have shown that the ClpXP machinery degrades 5_2_-knotted proteins from the UCH family with unprecedentedly slow kinetics and have attributed this to extremely high mechanostability due to the knotted structure^65^. Remarkably though, and in contrast to the earlier study, our results clearly establish that ClpXP can also degrade the more complex 5_2_-knotted protein UCH-L1 if the local stability of the protein near the degron is not too high (Fig. 5C) or if degradation is initiated from the N-terminus (Fig. 5B). We note that our experiments were performed in a buffer that did not contain monovalent cation whilst those of Hsu and coworkers used 100 mM KCl^65^, however, we have strong evidence that this is unlikely to be the origin of the differences observed. First, the results of our degradation assays on the same constructs (UCH-L1-ssrA) are the same as those from the Hsu group, second, when we destabilise the C-terminal region of the β-sheet by mutation we see efficient degradation even when degradation is initiated from the C-terminus.

Our results conclusively show that the 5_2_ knot in UCH-L1 does not confer mechanostability on the protein as degradation is efficient if initiated from the N-terminus or from the C-terminus if the local stability is decreased by mutation. This leads to the question of how the ClpXP machinery degrades this 5_2_-knotted protein. It is possible that, in the absence of a stable structured domain blocking the knot from falling off the end of the polypeptide chain, the 5_2_ knot simply slips along the chain until it drops off the end, Fig. 3B. These results suggest that, after unfolding of the UCH-L1 structure, the 5_2_ knot is sufficiently dynamic to move along the chain as translocation takes place or that the 5_2_ knot, although large, can enter the translocation pore.

Finally, we also studied an N-terminal fusion of the 5_2_-knotted UCH-L1 with the very stable ThiS domain and obtained similar results to those obtained for the ThiS-YbeA-ssrA. In this case, incomplete degradation was observed in addition to a degradation intermediate (referred to as I_TU_), Fig. S8. Mass spectrometric analysis of this intermediate revealed that it corresponded to the ThiS domain with a C-terminal tail of some 32–35 residues, Fig. S11. From these results, we can conclude that the tightened knot must be able to penetrate the ClpXP translocation pore as it has been estimated from optical tweezer studies on the mechanical unfolding of UCH-L1 that the 5_2_ knot comprises roughly 40 residues up to 40 pN of force and even if it is further tightened by higher forces only reduces to some 23-24 residues^33^. Thus, the observed tail of ThiS-UCH-L1-ssrA is not long enough to accommodate a 5_2_ knot, even if fully tightened. In fact, the tail is just long enough to span the distance from the ClpX opening to the ClpP proteolytic sites provided it is in an extended unfolded conformation. Therefore, the 5_2_-knot must either be able to pass through the translocation pore and be hydrolysed by the ClpP machinery, Fig. 6B, or it becomes stuck within the ClpXP machine but at least parts of the knotted regions are sufficiently close to the proteolytic sites to be hydrolysed, Fig. 6C.

The degradation of the knotted fusion proteins results in the formation of intermediates which have also been seen in the degradation of other multi-domain proteins^80–82^ and also fusions of the shallow trefoil-knotted MJ0366^64^. Here, we have demonstrated that the incomplete degradation of deeply-knotted trefoil fusions and also fusions of 5_2_-knotted proteins can result in the production of stable partially degraded intermediate states. It has been proposed that these processes can give rise to potential new biological activities^64,80–82^. It is interesting to speculate whether this might be the case for the knotted proteins studied here. Inspection of the Pfam database shows that there exist three architectures (3055 sequences) that have a deep trefoil-knotted MTase domain fused to another domain at either N- or C-termini (PF02590). Remarkably, there are 46 different architectures (2769 sequences) where the more complex 5_2_-knot found in the UCH family of deubiquitinating enzymes is fused to another structured domain (PF01088). These results raise the possibility that partial degradation of a knotted fusion protein may play a role in the regulation of biological activity of the additional domain.

To the best of our knowledge, this is the first experimental study of the degradation of a trefoil-knotted protein with a deep knot in its structure and also the first study of the degradation of a 5_2_-knotted protein by an ATP-dependent protease initiating degradation from both termini and also investigating the role of local stability versus knotted structures in determining degradation rates. Overall, our data suggest that the ClpXP machinery is easily able to degrade a deeply 3_1_-knotted protein and a 5_2_-knotted protein. It is possible that, in these cases, once the knotted protein has unfolded the knot simply slips along the polypeptide chain and falls off the free terminus, although our results on knotted fusion proteins have also shown that in the case of both 3_1_- and 5_2_-knotted proteins it is possible for the tightened knot to enter the translocation pore.

## Experimental Procedures

### Plasmid and protein preparation

A truncated form of ClpX lacking the N-terminal domain (referred to as ClpXΔN) was used in all experiments. Residues 1–62 of full-length *Escherichia coli* ClpX in plasmid pRSETA were deleted using PCR mutagenesis to construct ClpXΔN. His-tagged ClpXΔN was expressed in *E. coli* BL-21 (DE3) cells. Cultures were grown in 2xTY media until OD_600_ = 0.7, when the temperature was reduced to 20 °C before induction with 100 µM isopropyl-β-D-thiogalactoside (IPTG) overnight. The protein was purified using Ni-NTA affinity (Qiagen) followed by size-exclusion chromatography on a Superdex 75 column (GE Healthcare) in ClpX storage buffer (25 mM Tris pH7.6, 200 mM KCl, 2 mM EDTA, 1 mM DTT). His-tagged ClpP from *E. coli* was expressed from the pET21a plasmid in *E. coli* C41 (DE3) cells. Cultures were grown until OD_600_ = 0.8 and induced with 100 µM IPTG overnight at 26 °C. Purification was carried out essentially as described previously^83^, using Ni-NTA affinity and ion exchange chromatography on a monoQ column (GE Healthcare), before dialysis into ClpP storage buffer (50 mM Tris-HCl pH 7.6, 1 mM DTT, 0.5 mM EDTA, 100 mM KCl, 10% glycerol).

The ssrA sequence (ANDENYALAA) was added to the C-terminus of the protein substrates (UCH-L1 in pRSET; YbeA, ThiS and ThiSYbeA in pET17b) using PCR mutagenesis. All tagged substrate proteins were expressed in *E. coli* C41(DE3) cells.

The protocol for purification of His-tagged UCH-L1-ssrA was based on methods reported previously^30,31,52,84^ Bacterial cultures were grown until OD_600_ = 0.8 before induction (400 µM IPTG, 2.5 h, 37 °C). Collected cell pellets were resuspended in UCH-L1 lysis buffer (50 mM Tris-HCl pH 7.5, 300 mM NaCl, 1 mM EDTA, 1 mM DTT) with protease inhibitor. The cells were lysed using an Emulsiflex cell cracker, and soluble protein was purified by Ni-NTA affinity before size-exclusion chromatography on a Superdex 75 column in UCH-L1 storage buffer (50 mM Tris-HCl pH 7.6, 0.5 mM EDTA, 5 mM DTT).

YbeA-ssrA, YbeA-ThiS-ssrA and ThiS-ssrA were purified essentially as described in^20,21^. Cultures were grown until OD_600_ = 0.8 and induced with 400 µM IPTG for 5 h at 37 °C. Collected cell pellets were resuspended in YbeA lysis buffer (20 mM Tris-HCl pH 7.5, 200 mM KCl, 10% glycerol, 1 mM DTT) before cell cracking. For YbeA-ssrA, the soluble fraction of the cell lysate was loaded on a Q Sepharose FF column (GE Healthcare). As expected from the theoretical pI of YbeA-ssrA (pI = 6.84), the protein appeared in the flow-through. The flow-through was diluted in 50 mM Tris-HCl pH 8.7, 1 mM DTT and loaded on to a monoQ column. Unexpectedly, the protein did not bind the column but appeared in the flow-through. However, several contaminants were removed by this purification step. Finally, the flow-through was concentrated using spin concentrators (Vivaspin, GE Healthcare) before size-exclusion chromatography on a HiLoad 26/60 Superdex 75 column in YbeA storage buffer (50 mM Tris-HCl pH 7.5, 200 mM KCl, 10% glycerol, 1 mM DTT). For ThiS-YbeA-ssrA (theoretical pI = 5.70), cell lysate was diluted in 50 mM Tris-HCl pH 8.4, 50 mM KCl, 1 mM DTT, and loaded on to a Q Sepharose FF column and eluted with a gradient to 1 M KCl. Protein-containing fractions were diluted in 50 mM Tris-HCl pH 8.4, 1 mM DTT and purified further on a monoQ column with a shallow gradient to 1 M KCl. Finally, the protein was desalted on a HiLoad 26/60 Superdex 75 column in YbeA storage buffer.

For ThiS-ssrA, cell lysate was diluted in 50 mM Tris-HCl pH 7.5, loaded on a Q Sepharose FF column and eluted with a gradient to 1 M NaCl. Protein-containing fractions were pooled and run on a HiLoad 26/60 Superdex 75 column in ThiS storage buffer (50 mM Tris-HCl pH 7.5, 150 mM NaCl).

Protein purity was assayed by SDS-PAGE, and protein masses were confirmed by mass spectrometry (PNAC Facility, Cambridge). Protein concentrations were determined spectrophotometrically at 280 nm, using extinction coefficients calculated with the ProtParam tool (http://web.expasy.org/protparam/).

### Enzymatic assays

Degradation assays were performed in degradation buffer (25 mM HEPES-KOH pH 7.6, 5 mM MgCl_2_, 0.032% Igepal CA-630, 10% glycerol). A reaction containing 0.5 µM ClpX∆N_6_, 1 µM ClpP_14_ and an ATP regeneration system (4 mM ATP, 16 mM creatine phosphate, 0.32 mg/ml creatine kinase) was pre-incubated at 25 °C for 2 min. To start the reaction, pre-warmed substrate in degradation buffer was added. The degradation process was monitored by SDS-PAGE and subsequent densitometry analysis (ImageJ). The amount of ClpP at each time point was used as an internal standard to which the amount of remaining substrate protein was normalised. The data were plotted in GraphPad Prism and fitted to a single exponential. The data were normalized using the equation:

$${y}_{norm}=\frac{{y}_{t}-{y}_{\infty }}{{y}_{0}-{y}_{\infty }}$$

where y_t_ is the signal at time *t*, y_∞_ is the signal at infinite times and y_0_ is the initial signal at *t* = 0.

The initial rates of the reactions were calculated from the slope of the initial linear phase, taking into account the starting concentration of substrate and the total concentration of enzyme.

Although degradation assays were frequently repeated to ensure reproducibility, often the time points used were different and, therefore, calculations of errors difficult. However, the degradation assays and rates used in the calculation of Michaelis-Menten kinetics were repeated in triplicate under the same conditions. In this case, the largest errors were no more than 20% and frequently smaller than this, see Fig. 2B. For experiments on UCHL1-ssrA, repeats employed the same time points and here we have calculated the mean and the standard deviation, Fig. S12.

### Stability assays

Thermal stability assays were monitored by far-UV circular dichroism on a Chirascan spectrometer (Applied Photophysics). Protein samples (10 µM) in a 1 mm pathlength cuvette were heated from 20–90 °C in 2 °C steps with 2 min equilibration at each temperature. Ellipticity scans were performed between 220–240 nm, stepsize 1 nm.

### Mass spectrometry

Identification of gel band proteins by peptide mass fingerprinting using trypsin and/or LysC digestion and MALDI mass spectrometry was performed by the Protein & Nucleic Acid Chemistry Facility (PNAC), Cambridge.

### Size-exclusion chromatography

Analytical size exclusion chromatography (SEC) was performed on an S200 10/300 GL column (GE Healthcare). A 500 µL loop was used for sample loading, and the sample was eluted at a flow rate of 0.5–1 mL min^−1^. The gel phase distribution parameter, *K*_av_, was calculated as

$${K}_{av}=\frac{{V}_{e}-{V}_{0}}{{V}_{t}-{V}_{0}}$$

where V_e_ is the elution volume measured from the center of the eluted peak, V_0_ is the void volume of the column determined from the elution volume of Blue Dextran 2000 and V_t_ is the total volume of the column. A calibration curve was prepared using the proteins in the Low Molecular Weight Gel Filtration Kit (GE Healthcare). The *K*_av_-value of each protein standard was plotted against the logarithm of its molecular weight. The linear regression of this plot was used to determine the molecular weight of the YbeA-ssrA and ThiS-YbeA-ssrA.

### Chemical crosslinking of ssrA-peptide

A synthesised ssrA peptide was attached to a mutant of UCHL-1 where all natural cysteines had been removed and a single cysteine had been inserted in a position close to the N-terminus (referred to as UCH-L1 Q2C). Chemical crosslinking of the ssrA peptide to the cysteine was performed in a two-step reaction using the heterobifunctional crosslinker sulfo-SMCC (Pierce, Thermo Scientific). This crosslinker has an amine-reactive succinimidyl ester at one end and a sulfhydryl reactive maleimide group at the other, separated by a cyclohexane spacer arm. The ssrA-peptide (NH_2_-GGWDHHHHHHAANDENYALAA-COOH, Pepceuticals) was dissolved in 100 mM sodium phosphate, pH 6.5, 30 mM NaCl to a final concentration of 6.4 mM. The Sulfo-SMCC crosslinker was dissolved in ultrapure water to a final concentration of 30 mM. UCH-L1 Q2C (45 µM) in UCH-L1 storage buffer was reduced with 5 mM TCEP for 1 h at room temperature. The protein was then buffer exchanged by two passes through a 7 kDa cut-off spin desalting column (Zeba spin, Thermo Scientific) into PBS, 2 mM EDTA. Reaction 1 (conjugation of crosslinker to peptide) was carried out by mixing crosslinker in 10 times molar excess with peptide and incubating for 40 min at room temperature with agitation. Reaction 1 was quenched by adding glycine (100 mM final concentration). Excess unreacted crosslinker was removed from the peptide by chromatography on a column with a 1.8 kDa exclusion limit (Pierce, Thermo Scientific). Fractions containing peptide were identified by absorbance at 280 nm. Reaction 2 (conjugation of activated peptide-crosslinker to the protein cysteine) was carried out by mixing reduced protein with activated, purified peptide in 20 times molar excess before overnight incubation at 4 °C with rotation. Reaction 2 was quenched by addition of 5 mM DTT. Excess activated peptide was removed by desalting on a PD-10 column (GE Healthcare) into PBS, 1 mM DTT. Fractions containing the final ssrA-tagged protein were identified by measuring the absorbance at 280 nm and at 240 nm (indicative of the crosslinker), and by SDS-PAGE. The ssrA-tagged protein was separated from unreacted protein using Ni-NTA affinity chromatography.

## Supplementary information

Supplementary Information

## Data Availability

All materials and data are available from SEJ and LSI (sej13@cam.ac.uk and lsi10@cam.ac.uk).
