# Supplementary material for: The AAA+ protease ClpXP can easily degrade a 31 and a 52-knotted protein
Source: Sci Rep. 2019 Feb 20;9:2421. doi: 10.1038/s41598-018-38173-3 (PMC6382783; doi:10.1038/s41598-018-38173-3)
Supplement: Supplementary file 1 — Supplementary Information [file 41598_2018_38173_MOESM1_ESM.docx]

**The AAA+ protease ClpXP can easily degrade**

**a 3_1_ and a 5_2_-knotted protein**

**Elin M. Sivertsson, Sophie E. Jackson & Laura S. Itzhaki**

**SUPPLEMENTARY INFORMATION**

A
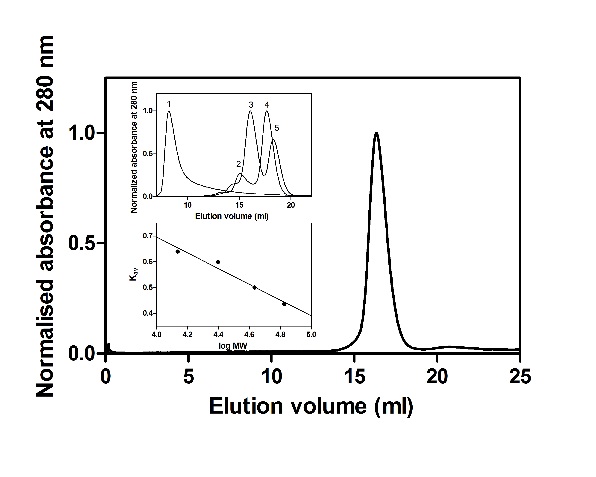


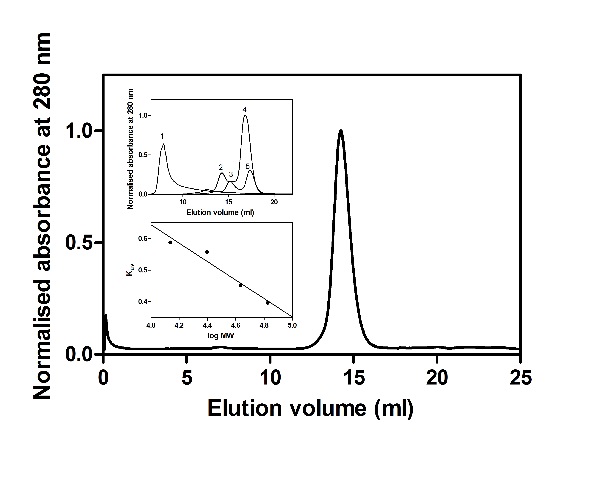


B

**Figure S1. Determination of the oligomeric state of knotted protein substrates by size-exclusion chromatography.** (A) YbeA-ssrA (12 µM), (B) ThiS-YbeA-ssrA (10 µM). Main: Elution profile. Inset, top: Elution profile of (1) Blue dextran (2000 kDa), (2) Albumin (67 kDa), (3) Ovalbumin (43 kDa), (4) Chymotrypsinogen A (25 kDa) and (5) RnaseA (13.7 kDa). Inset, bottom: Calibration curve.

**
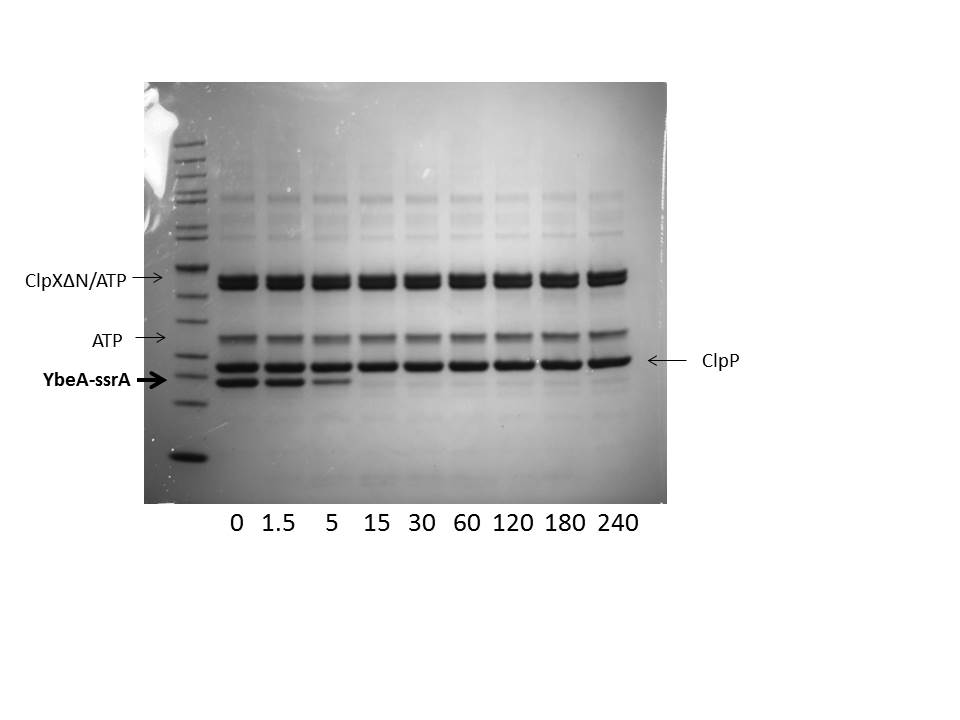
**

B

A

C**
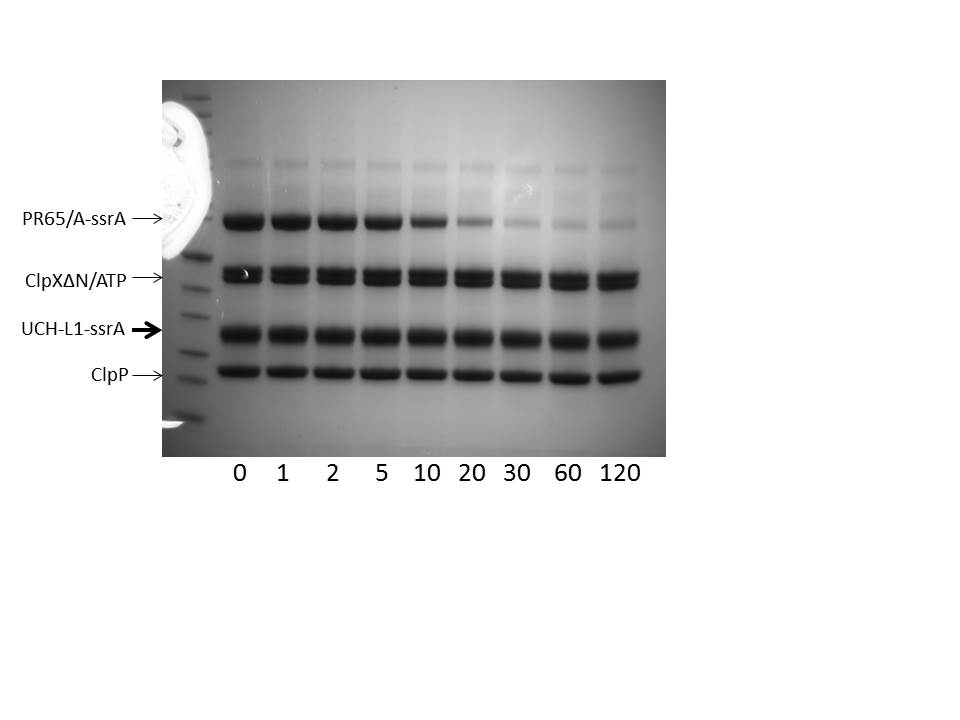
**


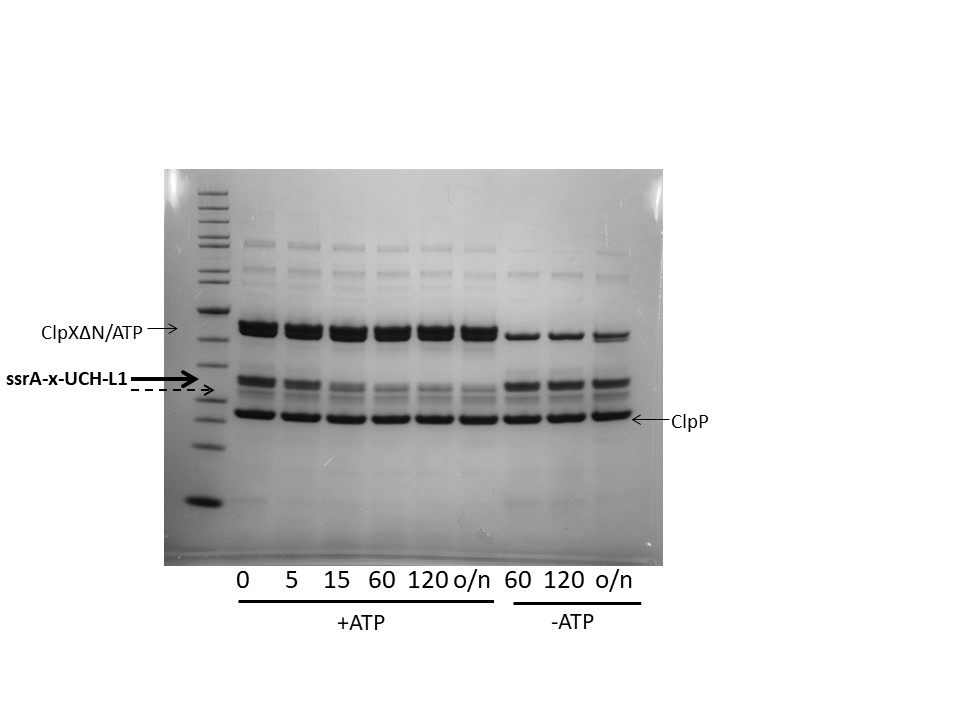


**Figure S2. SDS-PAGE data of knotted protein degradation assays.** (A) Degradation of 3_1_-knotted protein YbeA-ssrA. (B) Degradation competition experiment with equimolar amounts of UCH-L1-ssrA and PR65/A-ssrA (5 µM each). (C) Degradation of UCHL-1 with a crosslinked N-terminal ssrA-tag (ssrA-x-UCH-L1, 10 µM). Bold arrow marks the position of ssrA-x-UCH-L1 band. Dashed arrow marks the position of non-crosslinked UCH-L1 Q2C. Last three lanes show a control reaction without ATP. Times in minutes indicated at the bottom of the gels, o/n = overnight incubation. “ATP” denotes protein bands originating from the ATP regeneration system (creatine phosphate/creatine kinase).


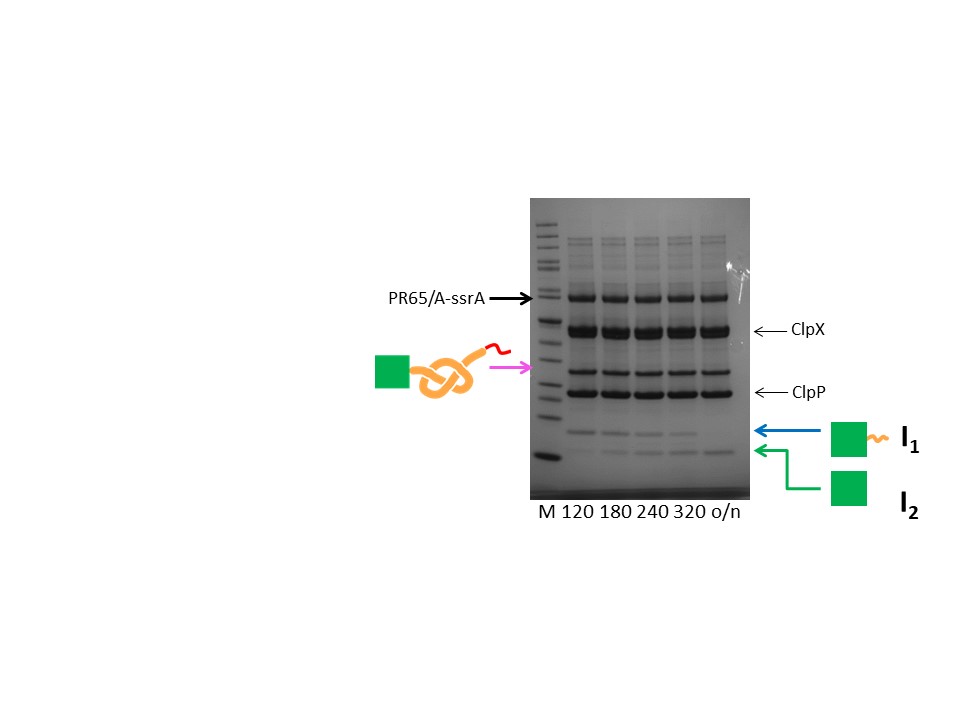
A


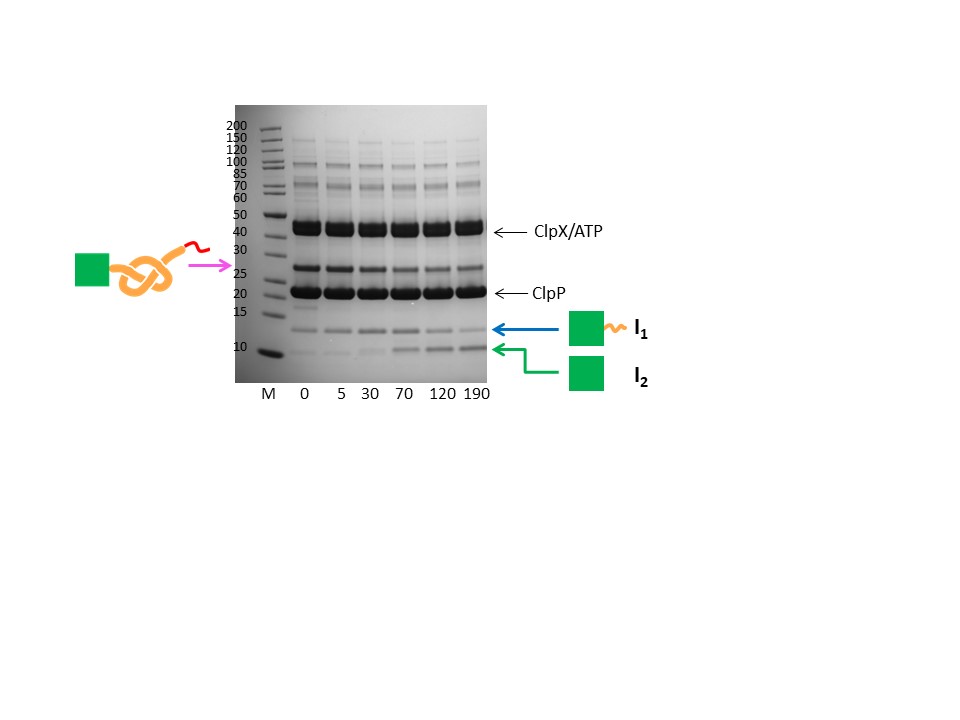
B

C

**Figure S3. Degradation of ThiS-YbeA-ssrA.**

(A) Gradual processing of intermediate I_1_ into intermediate I_2_ at later time points of degradation competition experiment with PR65/A-ssrA and ThiS-YbeA-ssrA. The first 120 minutes of the experiment is shown in Fig. 4C. The reactions contained 0.5 µM ClpXΔN_6_ and 1 µM ClpP_14_. (B, C) Degradation of ThiS-YbeA-ssrA (5 µM) with the concentration of ClpXP increased three times (1.5 µM ClpX∆N_6_, 3 µM ClpP_14_), monitored by (B) SDS-PAGE and (C) subsequent densitometry. All reactions contained 4 mM ATP with a regeneration system. The density of a protein band was normalised to the density of the ClpP band in the same lane. Times in minutes indicated below the gel, o/n = overnight incubation. M = marker in kDa. Full-length ThiS-YbeA-ssrA in pink, degradation intermediate I_1_ (ThiS + 37 residues) in blue, degradation intermediate I_2_ (ThiS) in green.

A

B
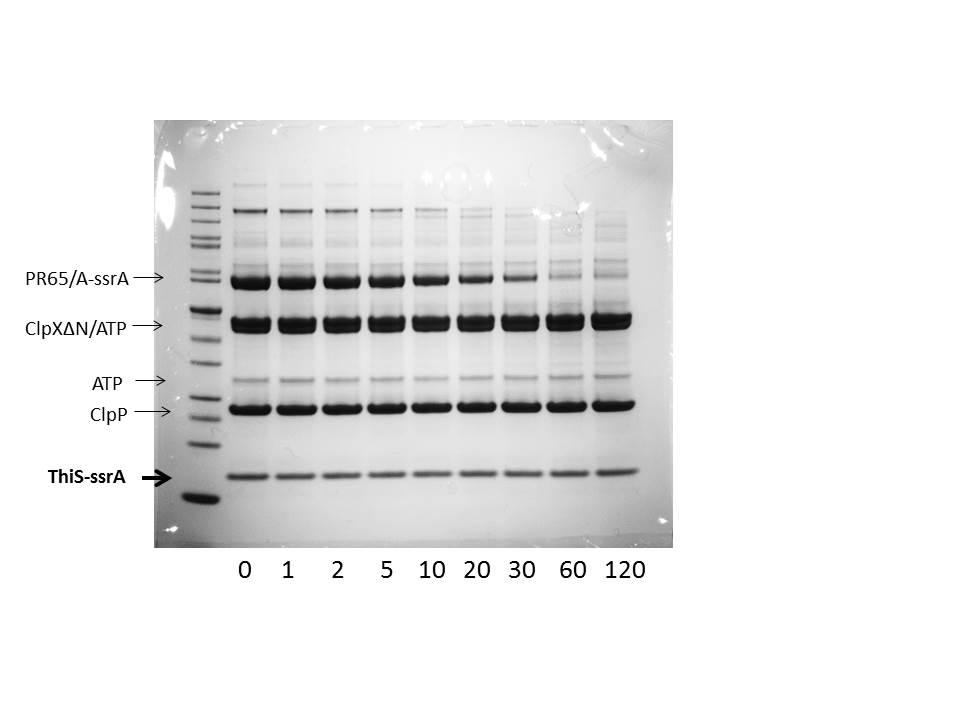

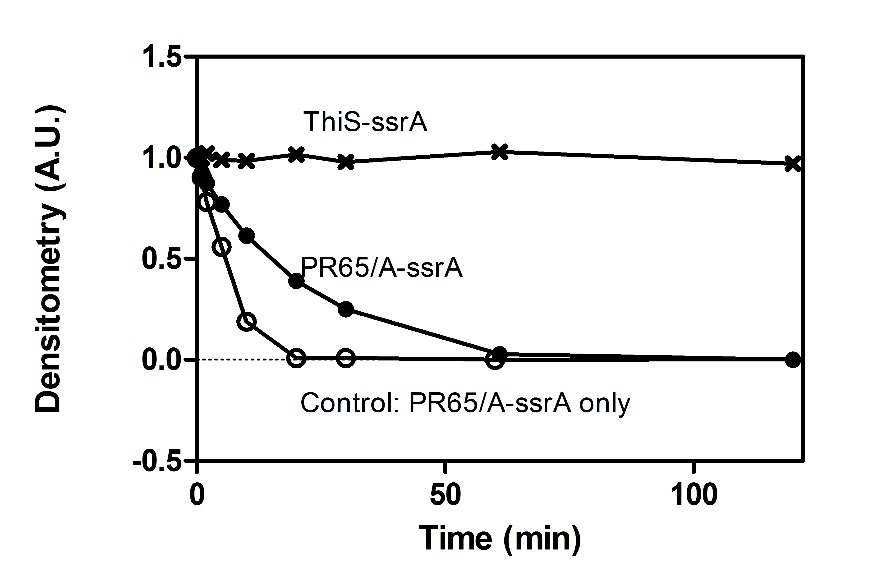


**Figure S4. Degradation competition experiment with equimolar amounts of PR65/A-ssrA** **(closed circles) and ThiS-ssrA (crosses)** (5 µM each), monitored by (A) SDS-PAGE and (B) densitometry. Control reaction with PR65/A-ssrA (5 µM) without competitor shown as open circles.

**
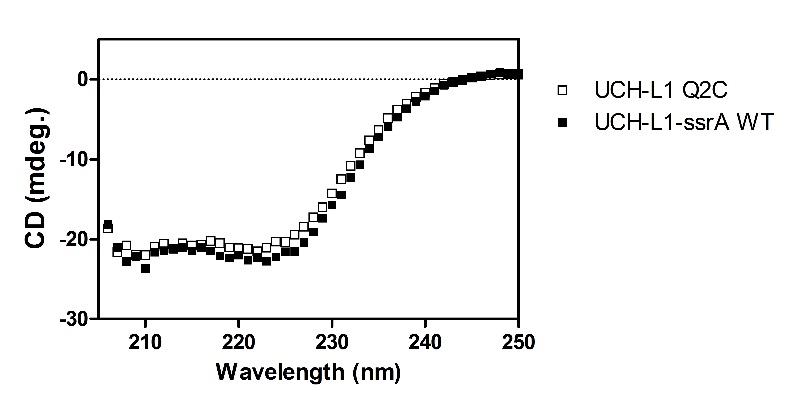

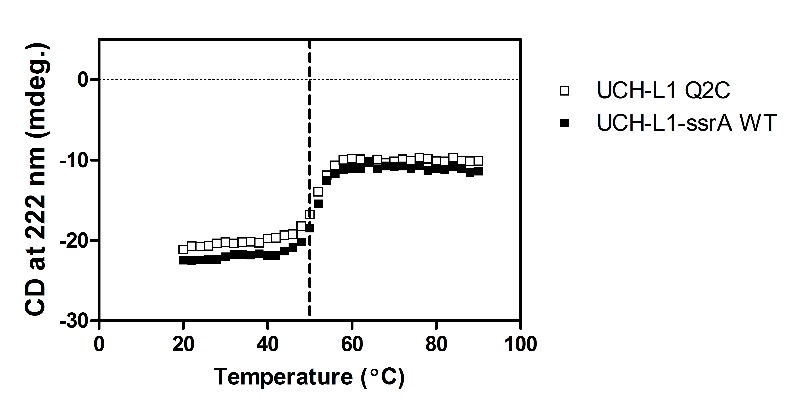
**

**C**

**A**

**B**

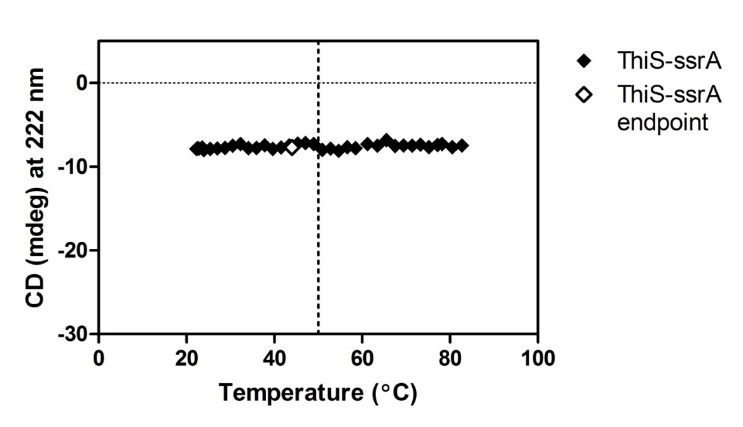


**D**

**D**

**Figure S5.** Thermal stability of substrate proteins (10 µM), measured by circular dichroism at 222 nm. UCH-L1 variants were in 50 mM Tris pH8, 150 mM Tris, 1mM TCEP. ThiS-ssrA was in 25 mM HEPES-KOH pH 7.6, 5 mM MgCl_2_, 1 mM DTE. The dashed line in (B)-(D) marks 50 °C, for ease of comparison. (A) UCH-L1-ssrA WT and UCH-L1 Q2C display the same native structure at 25 °C. (B) UCH-L1-ssrA WT and UCH-L1 Q2C show both show a single transition with the same midpoint (51 °C). (C) UCHL1 mutants showed different stabilities. (D) This-ssrA did not melt at the temperatures reached (up to 83 °C).


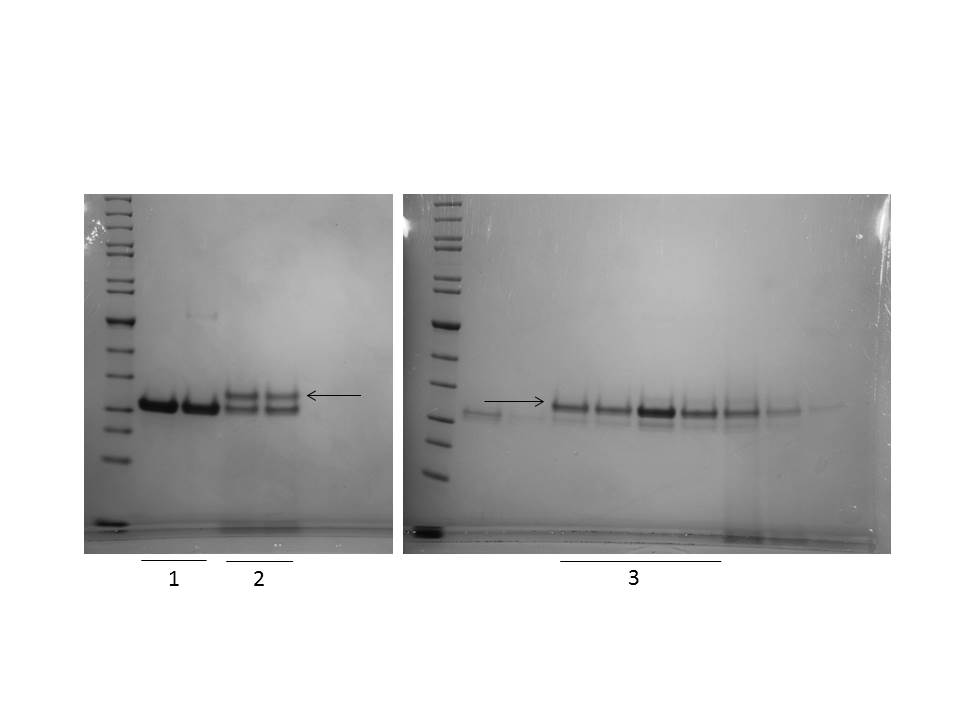


**Figure S6. Yield of crosslinking reaction between UCH-L1 Q2C and ssrA-peptide via the crosslinker sulfo-SMCC.** SDS-PAGE gels showing UCH-L1 Q2C before (lanes 1) and after crosslinking with ssrA-peptide (lanes 2) and after accumulation and purification by IMAC (lanes 3). Arrow marks the position of the final crosslinked product ssrA-x-UCH-L1.


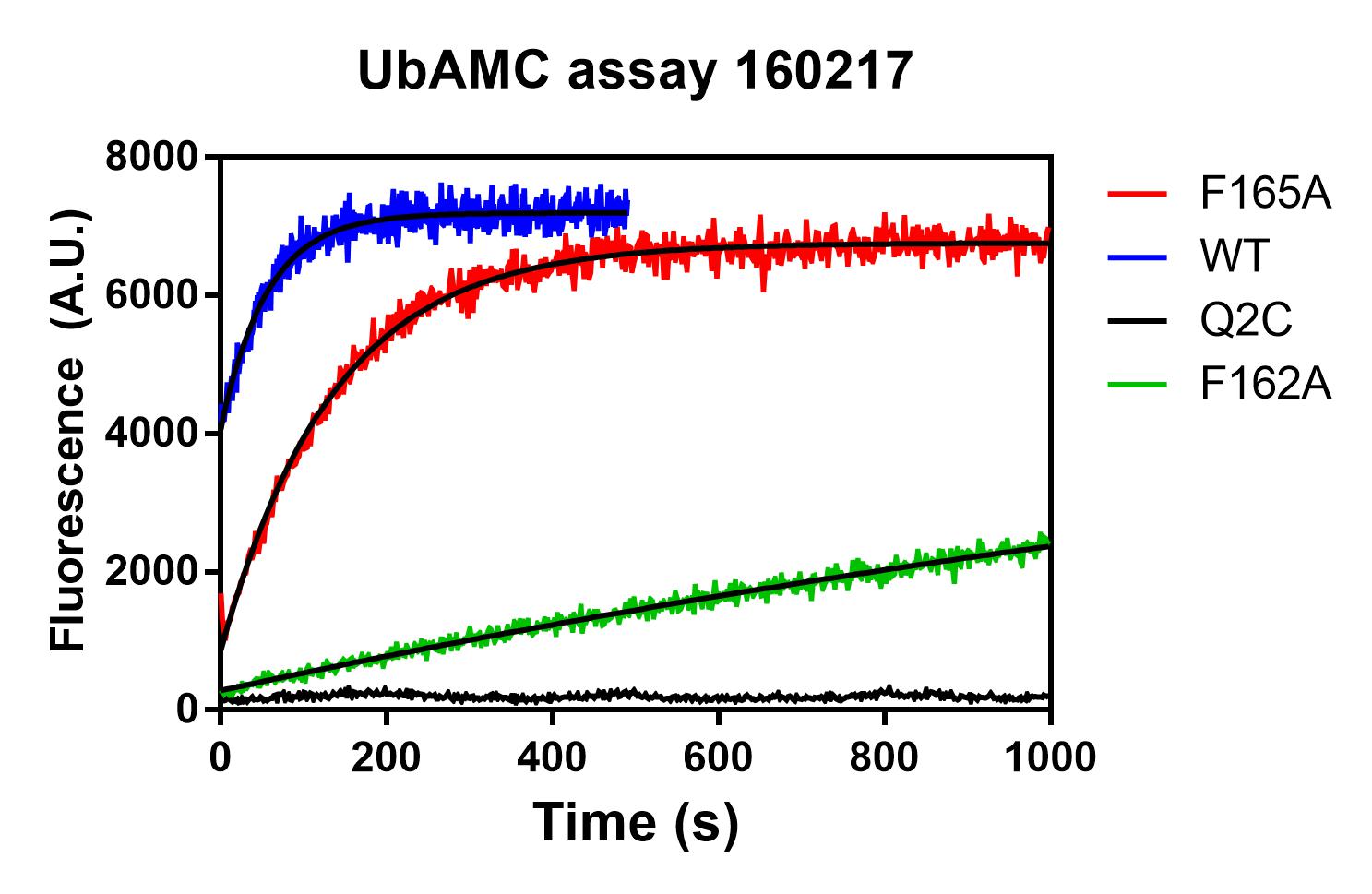


**Figure S7. Functional assay for UCH-L1 variants.**

Note that variant Q2C lacks enzymatic activity as the catalytic cysteine (Cys90) has been mutated to alanine.

**
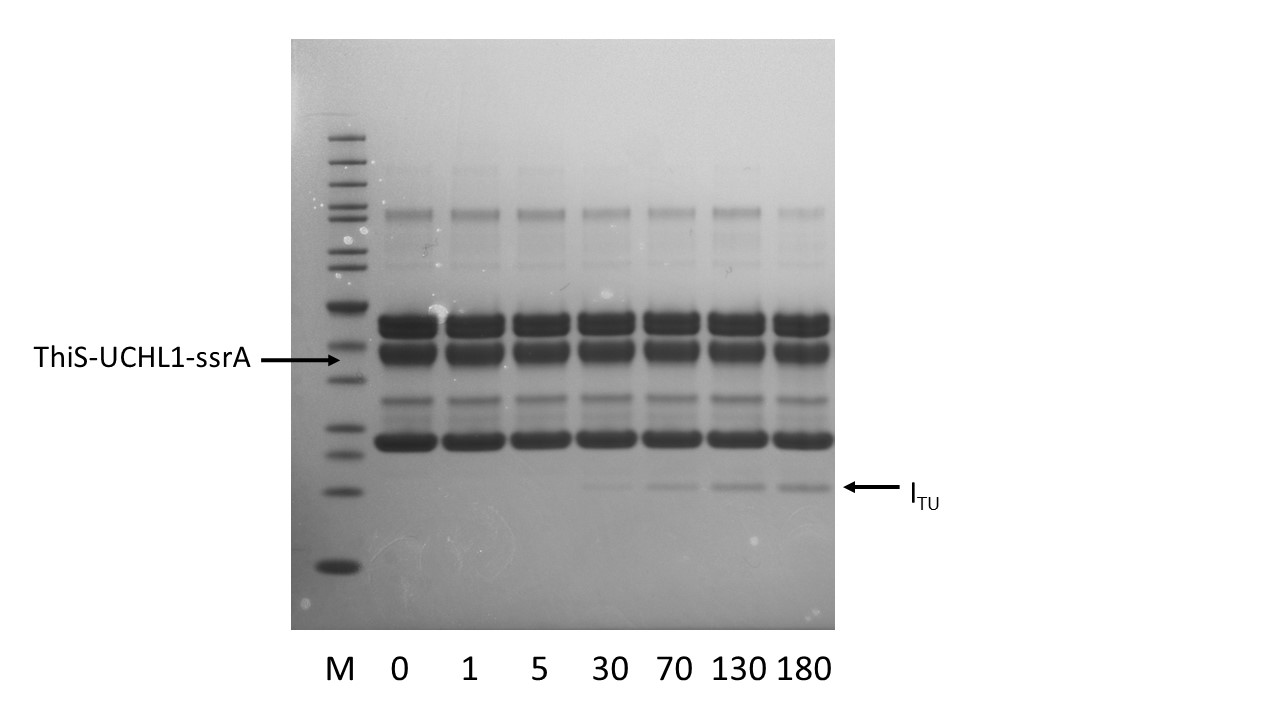
**

**
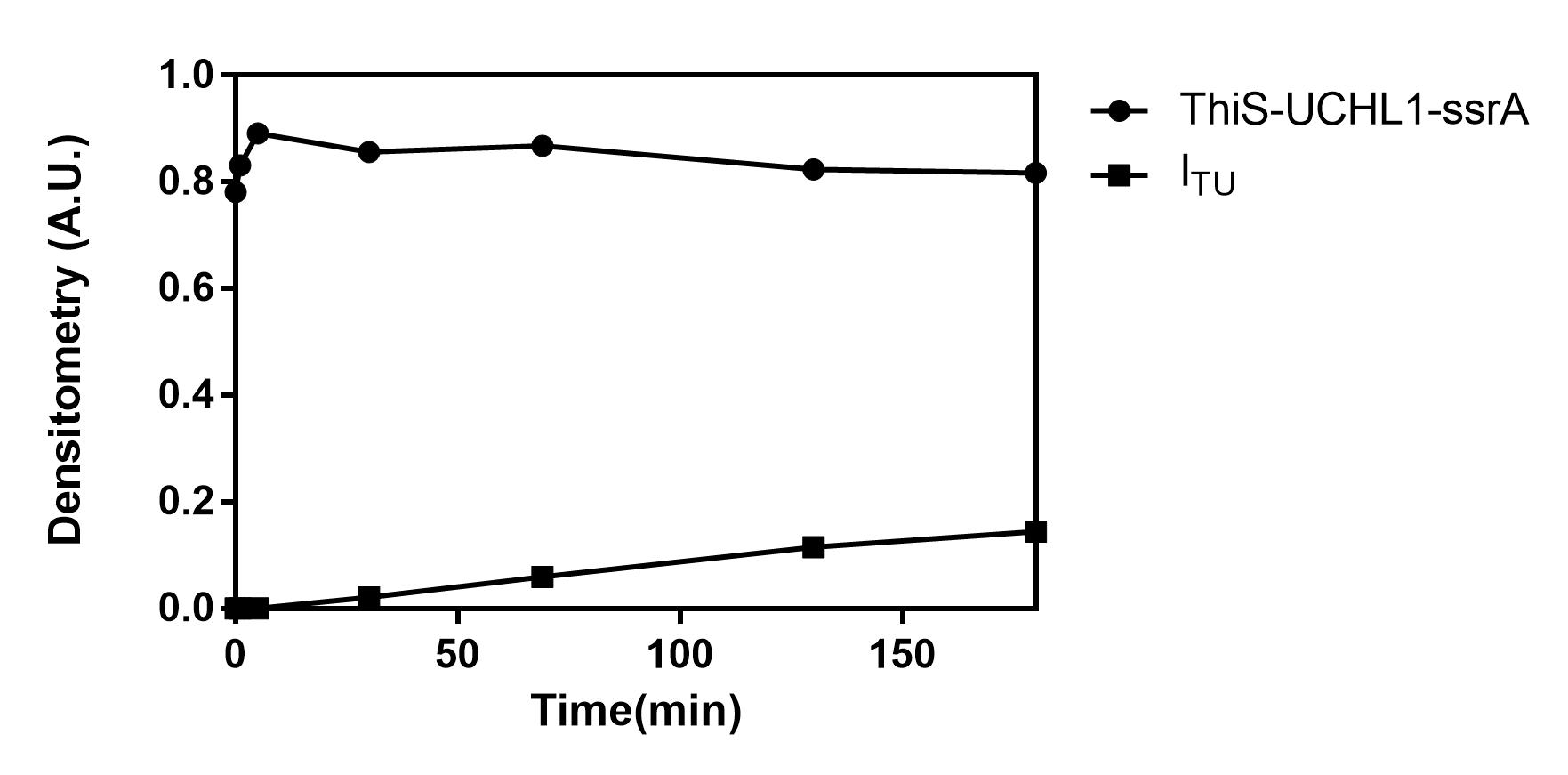
**

**FIG. S8. Degradation of ThiS-UCHL1-ssrA.** Degradation of ThiS-UCH-L1-ssrA (7.7 µM by ClpXP (0.5 µM ClpXΔN_6_, 1 µM ClpP_14_) monitored by SDS-PAGE (A) and densitometry (B). Closed circles: ThiS-UCH-L1-ssrA, closed squares: degradation intermediate I_TU._ N.B Arbitrary units are used in Panel B and the scaling for ThiS-UCHL1-ssrA band is not the same as that used for I_TU,_ in order to show the increase in amount of I_TU_ that accumulates with time. From the gel shown in Panel A it is clear that the amount of I_TU_ is considerably less than 5% of the amount of ThiS-UCHL1-ssrA.

**
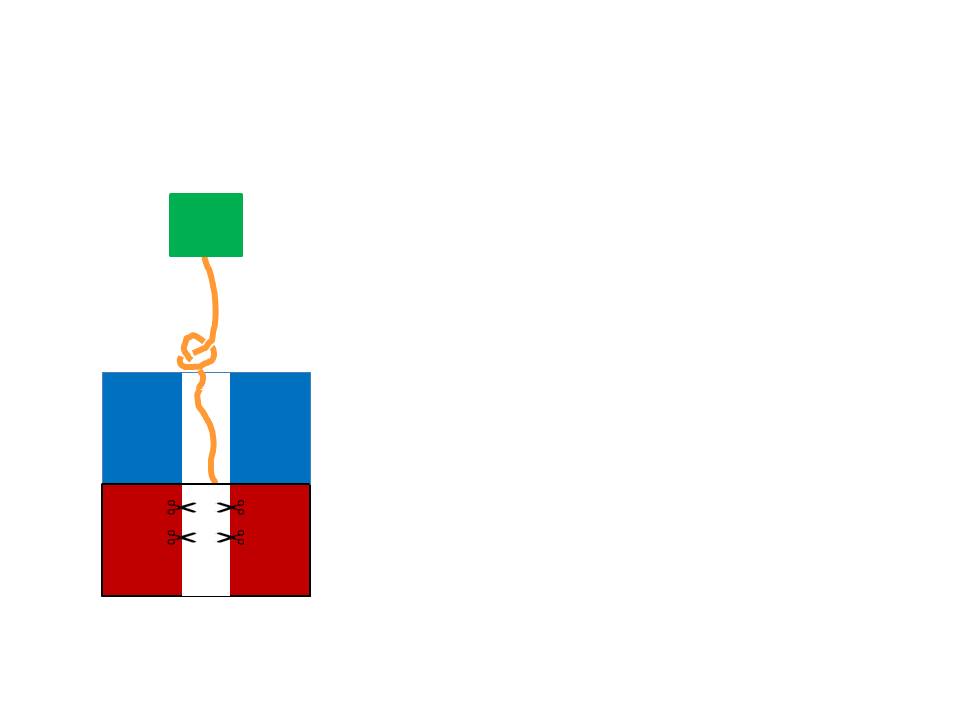
Figure S9 Alternative model for the degradation of the trefoil-knotted ThiS fusion protein**. Trefoil knotted protein (orange), ATP-dependent protease (blue), proteolytic domain (red with scissors), highly stable ThiS ’plug’ domain (green). In this mechanism the trefoil knot tightens before it has reached the stable ThiS domain.

**
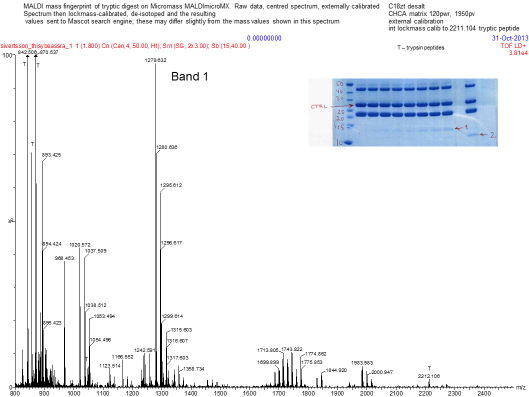
**A

**
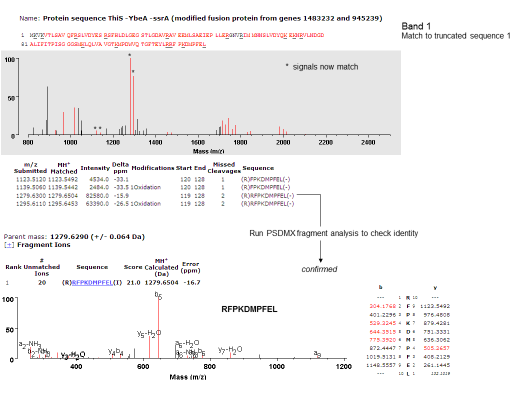
**B

**
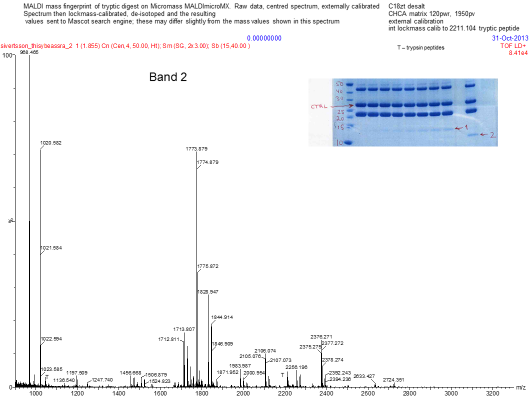
**C

**
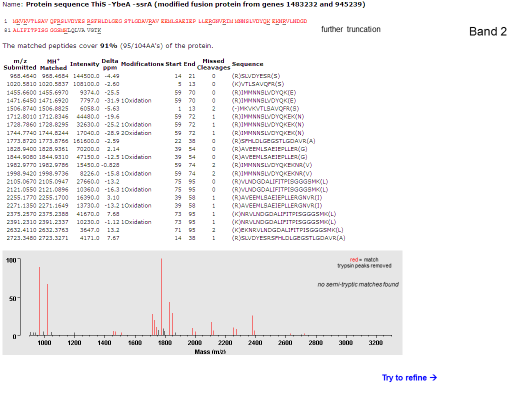
**D

E

**
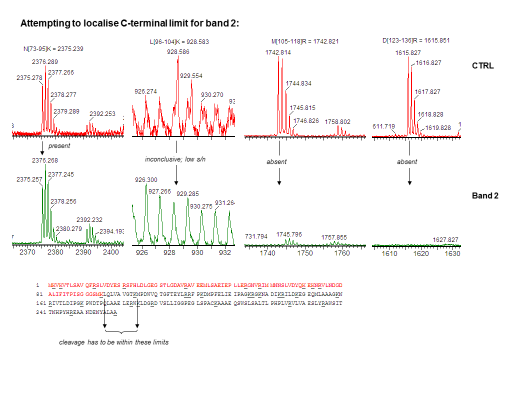
**

**Figure S10.** Mass spectrometric characterisation of the degradation intermediates from This-YbeA-ssrA fusion. (A) shows the MS data from the tryptic digest of band 1 corresponding to I_1_. (B) shows the match of the fragments from band 1 (I_1_) identified by MS to the ThiS-YbeA-ssrA sequence. (C) shows the MS data from the tryptic digest of band 2 corresponding to I_2_. (D) shows the match of the fragments from band 2 (I_2_) identified by MS to the ThiS-YbeA-ssrA sequence. (E) Analysis of determination of the C-terminal end of band 2 (I_2_) by MS and limits of confidence.

A


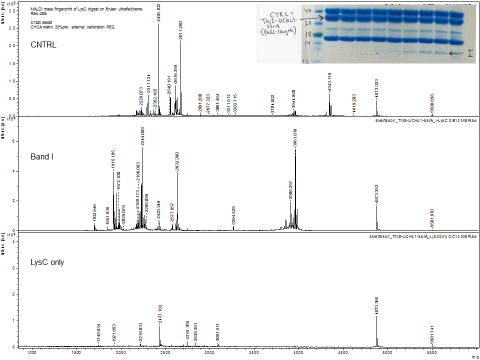


B

**
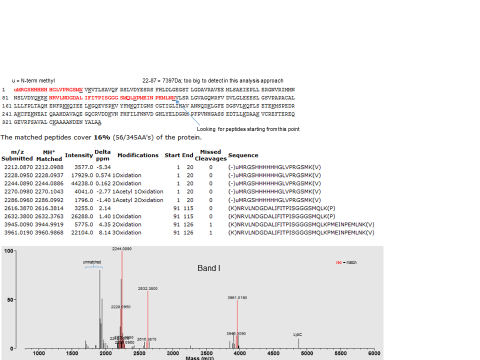
**

**
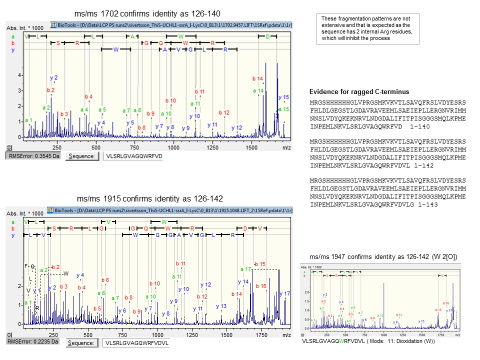
**C

**Figure S11.** Mass spectrometric characterisation of the degradation intermediates from This-UCH-L1-ssrA fusion. (A) shows the MS data from a LysC digest of the control (full-length ThiS-UCH-L1-ssrA), band 1 corresponding to I_TU_ and a LysC control. (B) shows the match of the fragments from band 1 (I_TU_) identified by MS to the ThiS-UCH-L1-ssrA sequence. (C) shows the MS data from the LysC digest of band 1 showing evidence that the C-terminal ends of the degradation product are ragged.

**Figure S12.** Analysis of the reproducibility and errors in the determination of the amount of uncleaved UCHL1-ssrA in the degradation assay from the densitometric analysis. Each data point shows the average calculated for the amount of full-length undegraded UCHL1-ssrA in solution over time for three separate experiments. The error bars shown are the standard deviation from the mean calculated from the three datasets. In each assay the amount of UCHL1-srrA was normalised against ClpXP concentration and was to 1 at t=0.
